# Unraveling the stepwise maturation of the yeast telomerase including a Cse1 and Mtr10 mediated quality control checkpoint

**DOI:** 10.1038/s41598-021-01599-3

**Published:** 2021-11-12

**Authors:** Anna Greta Hirsch, Daniel Becker, Jan-Philipp Lamping, Heike Krebber

**Affiliations:** 1 Abteilung für Molekulare Genetik, Institut für Mikrobiologie Und Genetik, Göttinger Zentrum für Molekulare Biowissenschaften (GZMB), Georg-August Universität Göttingen, Göttingen, Germany; 2 Present Address: Philipps-Universität Marburg, Klinik für Dermatologie Und Allergologie, Baldingerstraße, 35043 Marburg, Germany

**Keywords:** RNA quality control, RNA transport, Nuclear transport, Senescence

## Abstract

Telomerases elongate the ends of chromosomes required for cell immortality through their reverse transcriptase activity. By using the model organism *Saccharomyces cerevisiae* we defined the order in which the holoenzyme matures. First, a longer precursor of the telomerase RNA, *TLC1* is transcribed and exported into the cytoplasm, where it associates with the protecting Sm-ring, the Est and the Pop proteins. This partly matured telomerase is re-imported into the nucleus via Mtr10 and a novel *TLC1*-import factor, the karyopherin Cse1. Remarkably, while mutations in all known transport factors result in short telomere ends, mutation in *CSE1* leads to the amplification of Y′ elements in the terminal chromosome regions and thus elongated telomere ends. Cse1 does not only support *TLC1* import, but also the Sm-ring stabilization on the RNA enableling Mtr10 contact and nuclear import. Thus, Sm-ring formation and import factor contact resembles a quality control step in the maturation process of the telomerase. The re-imported immature *TLC1* is finally trimmed into the 1158 nucleotides long mature form via the nuclear exosome. TMG-capping of *TLC1* finalizes maturation, leading to mature telomerase.

## Introduction

The protection and maintenance of the ends of linear chromosomes during replication is crucial for dividing eukaryotic cells. Therefore, nucleoprotein structures called telomeres shield the ends of linear chromosomes from double strand break repair activities and thus from end-to-end ligations that would cause massive genome rearrangements and genome instability^1,2^. These telomeres are the target of a ribonucleoprotein (RNP) complex, called telomerase that protects the chromosome ends from progressive shortening by extending them through its reverse transcriptase activity^3^. While in most somatic cells telomere shortening occurs after sufficient cell divisions and causes a cell to enter into replicative senescence, stem cells, germ cells, lymphocytes and unicellular eukaryotes like yeast express telomerase to encounter this effect. Remarkably, almost 90% of all cancer cells have re-activated the telomerase to bypass their proliferation limit^1,4–6^. As the production of functional telomerase is crucial for telomere maintenance, it is important to understand its assembly and the order of its step-wise maturation process to realize the underlying control mechanisms that generate functional telomerase.

The telomerase is an RNP complex that contains a long non-coding RNA component, *TLC1* (telomerase *c*omponent 1) composed of 1158 nucleotides in yeast, which functions as template for the synthetic telomere repeats and as a scaffold for the protein components of the holoenzyme, providing the reverse transcriptase activity^2,7–9^ Like mRNAs and most lncRNAs, *TLC1* is transcribed in the nucleus by RNA polymerase II (RNAP II) and it is subsequently capped with a monomethyl guanosine (m^7^G) cap and polyadenylated like mRNAs^7,10^. However, in contrast to mRNAs, but very similar to snRNAs, Tgs1 generates a 2,2,7-trimethylguanosine (TMG)-cap on *TLC1* in the nucleolus, which persists in the mature telomerase^7,11,12^. Interestingly, *TLC1* was described to exist in two forms in the cell, a 1158 nucleotides long mature form and a ~ 1300 nucleotides long precursor. The majority of the *TLC1* molecules that are present in cells is the short form and only ~ 10% of the *TLC1* RNA is the long polyadenylated form^13^. This might reflect that approximately 10% of the *TLC1* RNAs might be in their maturation phase and 90% are already matured. However, in addition to the cleavage and polyadenylation factor mediated transcription termination (CPF-CF), as used for mRNAs, a study uncovered the ability of the Nrd1-Nab3-Sen1 (NNS) system to terminate *TLC1* transcription and it was suggested that this would immediately generate the ~ 1150 nucleotides short form, characteristic of the mature telomerase^14,15^. If really both termination sites are equally used or if one is preferred over the other under certain conditions is currently unclear. Regardless of the transcription termination pathway used, the primary transcript is always longer than the mature form. Clearly, some of the *TLC1* molecules receive a ~ 80 nucleotides long poly(A) tail after transcription, which is removed during the maturation process and the exosome continues the trimming of the pre-*TLC1* RNA to 1158 nucleotides^7^.

In addition to these RNA-specific structural maturation events, *TLC1* provides a scaffold for several proteins^16^. In analogy to the snRNAs, the 3’-end of *TLC1* is bound by a heptameric ring, composed of seven Sm-proteins, that encircles and stabilizes *TLC1*^12,17,18^. Additionally, structurally important proteins as well as regulatory and catalytic factors bind to the *TLC1* RNA. One of the stem loops that are formed by *TLC1* is bound by the heterodimer Yku70-Yku80, which is important for a persisting nuclear localization through attachment of the telomerase to chromosome ends^19,20^. The central domain of *TLC1,* which includes the template domain for reverse transcription, is bound by the catalytic protein subunit Est2 and two accessory factors Est1 and Est3^2,7^. The Est proteins are stabilized by the Pop1, Pop6 and Pop7 proteins, which are also central components in the RNase MRP and the RNase P^21–23^.

Besides *TLC1* processing and the protein loading onto this scaffold, this RNA undergoes nucleo-cytoplasmic shuttling^7,24^. It is exported into the cytoplasm via Mex67-Mtr2 and Xpo1/Crm1^24^. On mRNAs and snRNAs the RNA-contact of Mex67 is mediated by the guard proteins Npl3, Gbp2 and Hrb1^25–28^. Thus, a similar mechanism is conceivable for *TLC1*. For Xpo1/Crm1 a physical RNA contact was shown via the cap binding complex (CBC) that interacts with the monomethyl cap^25^.

After loading of the Est proteins and the Sm-ring in the cytoplasm, *TLC1* is re-imported into the nucleus via Mtr10^24,29,30^. Prevention of shuttling, either by mutations in the export factors or the import factor results in telomere shortening defects and reduced *TLC1* levels^24,30,31^.

For snRNAs the transfer to the cytoplasm was shown to be crucial for the generation of a functional spliceosome, because when not exported, the longer, immature pre-snRNAs can be incorporated into the spliceosome and jeopardize splicing^25^. Thus, the immediate cytoplasmic drain of these noncoding RNAs is crucial for cell survival and might also be similarly important for the transcribed immature *TLC1*.

Interestingly, maturation events such as trimming and TMG-capping were suggested to be nuclear events that occur prior to *TLC1* shuttling and current models suggest that only the Est proteins and the Sm-ring are loaded in the cytoplasm^24,30,31^. Therefore, we investigated the maturation steps of *TLC1* in detail and uncovered a stepwise process. We found that pre-*TLC1* shuttles into the cytoplasm for the loading of the Sm-ring, the Est- and the Pop proteins. The subsequent re-import of the protein bound *TLC1* precursor not only requires Mtr10, but also Cse1, a novel import receptor for *TLC1.* Defects in *CSE1* result in amplified Y′ elements and thus elongated telomere ends and destabilized Sm-ring association on pre-*TLC1*. Thus, proper nuclear re-import of protein-bound pre-*TLC1* can only occur in the presence of both, intact Cse1 and Mtr10, which resembles a quality control step in the life cycle of the telomerase RNA. We further show that trimming of *TLC1* to the 1158 nucleotides short form is carried out by the nuclear exosome after shuttling. In a final step, TMG-capping of *TLC1* completes maturation. In summary we have uncovered that the maturation of *TLC1* occurs in a different order than anticipated and includs a cytoplasmic quality control step to ensure proper telomere function.

## Results

### The Sm-ring is loaded to the precursor of *TLC1* in the cytoplasm

**Figure 1:**
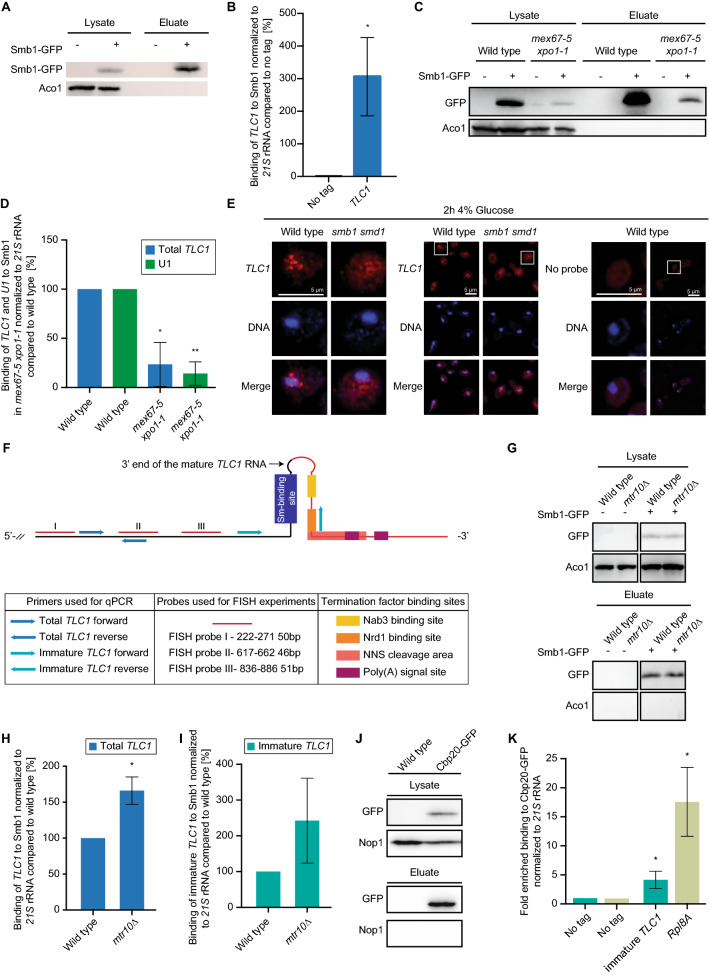
The longer precursor of *TLC1* binds the Sm-ring in the cytoplasm. (**A**) RIP experiments were carried out with GFP-tagged Smb1 from wild type cells. One example IP of Smb1 is shown on western blots. Aco1 served as a negative control. Images were cropped from the original. Original image can be found in Supplementary Fig. S1A. (**B**) Smb1 binds to *TLC1*. The Smb1 co-precipitated RNA was used as a template in qPCRs for *TLC1*. For this (and all following IPs carried out in this study), the amount of the eluted RNA was related to the RNA present in each lysate. Normalization to the mitochondrial *21S* rRNA is indicated on the y-axis. *n* = *4*. (**C**) A western blot shows an example IP of the precipitated Smb1 protein from the RIP experiment shown in (**D**). Images were cropped from the original. Original image can be found in Supplementary Fig. S1C. (**D**) Smb1 cannot bind to *TLC1* when its nuclear export is prevented. The Smb1 co-precipitated RNA was used as a template in qPCRs for *TLC1* in the indicated strains, shifted to 37 °C for 1 h. *n* = *3*. (**E**) *TLC1* accumulates in the cytoplasm of cells in which the Sm-ring is not properly assembled. FISH-experiment of a Cy3-labeled probe (red) targeting *TLC1* is shown in wild type cells and a strain in which the Sm-ring is not assembled trough mutation of *SMB1* and *SMD1* after glucose induced expression repression. The DNA is stained with DAPI. *n* = *3* (**F**) Scheme of the ~ 1300 nucleotides long immature *TLC1* and the primer positions for the amplification of the total and the immature *TLC1* molecules. (**G**) Western blot showing an example of a Smb1 IP used in (**H**) and (**I**) in RIP experiments. Images were cropped from the original. Original image can be found in Supplementary Fig. S1B. (**H**) Blocking nuclear re-import of *TLC1* increases the amount of the total *TLC1*. RIP experiments and subsequent qPCRs were carried out to show the total *TLC1* in the indicated strains. *n* = *3* (**I**) Blocking nuclear re-import of *TLC1* increases the amount of the immature *TLC1*. RIP-experiments were carried out and subsequent qPCR results of the immature *TLC1* in the indicated strains is shown. *n* = *3* (**J**) Western blot showing an example of a Cbp20 IP used in (**K**) for RIP experiments. Images were cropped from the original. Original image can be found in Supplementary Fig. S1D. (**K**) Immature *TLC1* binds to Cbp20. The Cbp20 co-precipitated RNA was used as a template in qPCRs for immature *TLC1. RPL8A* served as positive control. *n* = *3*.

Sm-ring binding to *TLC1* is a prerequisite for proper processing of this non-coding RNA, while the contact of the TRAMP-complex and the nuclear exosome prior to the Sm-ring attachment rather initiates full degradation of the transcript^17,18^. On snRNAs, Sm-ring loading was recently identified to be a cytoplasmic event^25^. It was shown that newly transcribed snRNAs are immediately exported from the nucleus to prevent an incorporation of the precursor RNAs into the spliceosome, which jeopardizes splicing^25^. *TLC1* receives its Sm-ring also in the cytoplasm (Fig. 1 and Ref.^30^). To analyze to which form the Sm-ring is loaded, the short 1158 nucleotides long mature form or the longer premature form, we did the following experiments. First, we extracted GFP-tagged Smb1 from cell lysates (Fig. 1A) and analyzed in RNA-co immunoprecipitation (RIP) experiments the general binding of *TLC1*. Subsequent qPCR experiments showed a clear binding of *TLC1* to Smb1, which was normalized to no tag as a negative control and related to *21S* rRNA (Fig. 1B). Importantly, when we repeated the experiment in the export mutant *mex67-5 xpo1-1* after shifting the cells for 2 h to the non-permissive temperature, which prevents the export of newly synthesized *TLC1* molecules, we detected a significantly decreased binding between the Sm-ring component Smb1 and *TLC1* (Fig. 1C,D). The decreased binding between Smb1 and U1 served as a control and is in agreement with published data^25^. These results suggest that the inhibition of the nuclear export of *TLC1* prevented the loading of the Sm-ring.

This finding is further supported by a fluorescense in situ hybridization (FISH) experiment targeting the *TLC1* RNA with a Cy3-labeled probe. *TLC1* is mostly detectable in the nucleus of wild type cells (Fig. 1E) and Ref.^24^. However, in a double mutant of *smb1 smd1*, which has a defective Sm-ring^32^, *TLC1* mis-localizes to the cytoplasm (Fig. 1E). These findings confirm that Sm-ring loading to *TLC1* occurs in the cytoplasm, which was also shown earlier via an inducible *TLC1-*RNA tagging experiment and is very similar to the cytoplasmic Sm-ring loading onto the snRNAs^25,30^. Therefore, we investigated the interaction between the Sm-ring and *TLC1* in the import receptor mutant *mtr10∆*. RIP-experiments show an increased binding of total *TLC1* (Fig. 1G–I). To get information whether the mature or the immature form of *TLC1* accumulates, we also investigated the binding of the precursor of *TLC1* to Smb1. A scheme of *TLC1* shows that the primers amplifying the unprocessed variant of *TLC1* will amplify any generated pre-*TLC1*, no matter if NNS or CPF-CF terminated (Fig. 1F).

Indeed, as shown in Fig. 1G–I, the binding of the immature *TLC1* to Smb1 increased even more in *mtr10∆* than that of the total *TLC1*, suggesting that rather the immature form accumulates. These findings indicate that the Sm-ring is loaded in the cytoplasm to the immature pre-*TLC1* and that this form waits for nuclear re-import.

Futhermore, it was shown that the export of *TLC1* requires Xpo1 in addition to Mex67^24^. For snRNA export it was shown that Xpo1 contacts the RNA via the cap binding complex (CBC)^25^. Since pre-*TLC1* contains an m^7^G cap, it seems likely that this 5’ cap is also bound by CBC. However, this has not been shown so far. Therefore, we carried out RIP experiments with Cbp20 showing that the immature form of *TLC1* associates with this CBC-component (Fig. 1J–K). Together, these findings suggest that pre-*TLC1* is exported via CBC-bound Xpo1.

### Cse1 is a novel nuclear re-import factor for *TLC1*

**Figure 2:**
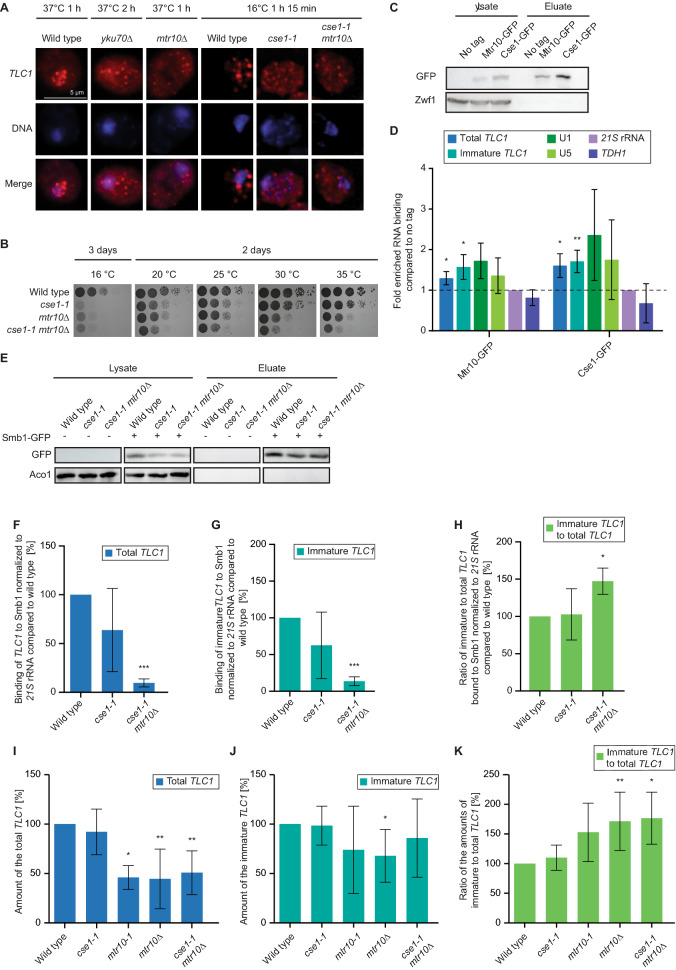
Cse1 is a novel nuclear import factor for *TLC1*. (**A**) *TLC1* mis-localizes to the cytoplasm in *cse1* mutants. A FISH-experiment for *TLC1* (red) is shown in the indicated strains that were shifted to the indicated non-permissive temperatures. *n* = *3.* Overview of the cells is shown in Supplementary Fig. S2A and negative controls in Supplementary Fig. S2B,C. (**B**) *CSE1* and *MTR10* genetically interact. Serial dilutions of the indicated strains were spotted onto full medium agar plates and incubated at the indicated temperatures. (**C**) RIP experiments were carried out with GFP-tagged Mtr10 and Cse1 from wild type cells. One example IP is shown on a western blot. Zwf1 served as a negative control. Images were cropped from the original. Original image can be found in Supplementary Fig. S2D. (**D**) Cse1 binds to *TLC1*. The Cse1 co-precipitated RNA was used as a template in qPCRs for total *TLC1*. *n* = *4* Mtr10 served as a positive control. *n* = *4* (**E**) RIP experiments were carried out in the indicated mutant strains. A western blot of an example IP is shown. Aco1 served as a negative control. Images were cropped from the original. Original image can be found in Supplementary Fig. S2E. (**F**) Binding of total *TLC1* to Smb1 decreases in *cse1-1* and *cse1-1 mtr10*∆ cells. RIP experiments and subsequent qPCRs were carried out. *n* = *4.* (**G**) Binding of immature *TLC1* to Smb1 increases in *cse1-1* and *cse1-1 mtr10*∆ cells. RIP experiments and subsequent qPCRs were carried out. *n* = *4.* (**H**) The ratio of the Smb1-bound immature versus total *TLC1* increases in *cse1-1 mtr10∆.* The ratios were calculated from the experiments shown in F and G. *n* = *4.* (**I**–**K**) The immature form of *TLC1* accumulates in nuclear re-import mutants. The RNA was isolated from the indicated strains after a 1 h 15 min temperature shift to the non-permissive temperature. Subsequent qPCRs were carried out with primers that detect either all *TLC1* forms (total *TLC1*) (**I**) or only the unprocessed form (immature *TLC1*) (**J**). (**K**) The ratio of immature to total *TLC1* increases in the import mutants. The ratios were calculated from the experiments shown in (**I**) and (**J**). *mtr10-1 n* = *3. cse1-1 n* = *6, mtr10∆ n* = *7* and *cse1-1 mtr10∆ n* = *5*.

Nuclear re-import of *TLC1* requires the nuclear import receptor Mtr10^29^. Interestingly, snRNAs are re-imported via Mtr10 and Cse1, another member of the kayopherin transport factor family^25^. Interestingly, Cse1 was originally identified as a nuclear export factor for the import adapter protein importin α, encoded by *SRP1*^33,34^. However, recently we have shown that in addition to its exporting function, Cse1 supports the import of Sm-ring-containing snRNAs, which thus resembles an important control step for its maturation, because the importer only interacts with Sm-ring containing snRNAs^25^. As the Sm-ring is also loaded onto *TLC1* in the cytoplasm, it seems reasonable to assume that Cse1 would also participate in its nuclear re-import. Therefore, we localized *TLC1* in the *cse1-1* mutant that was shifted to 16 °C, as it is cold sensitive. We found that while *TLC1* localized mainly to the nucleus in wild type cells, it was distributed throughout the cytoplasm in the *cse1-1* mutant (Fig. 2A, Supplemental Fig. S1), similar to *mtr10*∆ and *yku70*∆ strains (Fig. 2A) and Refs.^24,29^. Since Mtr10 and Cse1 both contribute to the nuclear import of *TLC1* and snRNAs, we created a double mutant and assayed its growth. Although the single mutants already showed growth defects in comparison to wild type, the double mutant grew even less, indicating a genetic interaction between these import receptor genes (Fig. 2B). Subsequent FISH experiments with the double mutant also showed a cytoplasmic accumulation, similar to that observed in the single mutants (Fig. 2A).

To verify binding of *TLC1* to Cse1, we carried out RIP experiments, in which we precipitated GFP-tagged versions of the import receptors Cse1 and Mtr10, the latter of which served as a positive control (Fig. 2C,D). We found that the binding of *TLC1*, most likely its immature form to both import receptors was significantly enriched, similar to the binding of the snRNAs U1 and U5 (Fig. 2D) and Ref.^25^. A 1.5 to twofold increase of the binding, although significant, might on first sight not seem to be very strong, but considering that the binding of both import receptors depends on the small GTPase Ran the low value is understandable. For import-cargo complex formation Ran must be in its GDP bound state. This is favored in the cytoplasm by the presence of the GTPase activating protein RanGAP1 and its co-factor RanBP1^35–37^. Cargo release from the import receptors occurs in the nucleus where RanGTP is present and the nucleotide exchange on Ran occurs with the nuclear exchange factor Prp20 (human RCC1)^37^. Upon cell lysis for the RIP experiment, both compartments get mixed up and pre-formed nuclear import complexes are attacked and disassembled through RanGTP. An additional difficulty is the low copy number of *TLC1* with ~ 10 to ~ 30 copies per haploid cell^7, 38,39^. However, despite these difficulties, we were able to detect binding of *TLC1* to both import factors, Mtr10 and Cse1 (Fig. 2C,D).

Analogous to *mtr10∆* (Fig. 1H,I) we investigated the interaction of *TLC1* and the Sm-ring in *cse1-1* and the *cse1-1 mtr10∆* double mutant. Interestingly, in contrast to *mtr10∆* where we observed an increased protein-RNA interaction, defective *cse1* leads to a decreased *TLC1*-Sm-ring binding for both, immature and mature *TLC1*, suggesting a stabilizing function of Cse1 for Sm-ring formation. Reduced Sm-ring formation in *cse1-1* did not affect the relative level of *TLC1* (Fig. 2I–K), suggesting that pre-*TLC1* might still be protected by other proteins. In the double mutant, in which both import receptors are missing, the Sm-ring binding is even further reduced to ~ 15% (Fig. 2E–G). In contrast to *cse1-1*, mutation in *MTR10* lead to reduced cellular levels of *TLC1* to about 50%, which is also the case in the double mutant *cse1-1 mtr10∆* (Fig. 2I–K). Our data suggest that although *TLC1* is mis-localized to the cytoplasm in both import factor mutants and both factors contribute to the re-import of this RNA, their functions differ in some aspects. Mtr10 seems to be the main import factor, as Sm-ring bound RNA accumulates in *mtr10∆* and thus reduces the overall level of *TLC1* (Fig. 1H,I). In contrast to this, Cse1 seems to stabilize the Sm-ring formation on pre-*TLC1* and mutants therefore rather delay maturation and in turn nuclear re-import. Remarkably, when we compare the binding of the Sm-ring to the immature *TLC1* in the different mutants, we still detect increased amounts (Fig. 2H), suggesting that this form is trapped in the cytoplasm and that Cse1 participates in nuclear import.

Together, these findings identify Cse1 as a novel import receptor that is also required for Sm-ring loading for *TLC1* and show that optimal nuclear re-import of this a large RNP relies on more than one import receptor.

### Mutation of CSE1 leads to amplified Y′ elements

**Figure 3:**
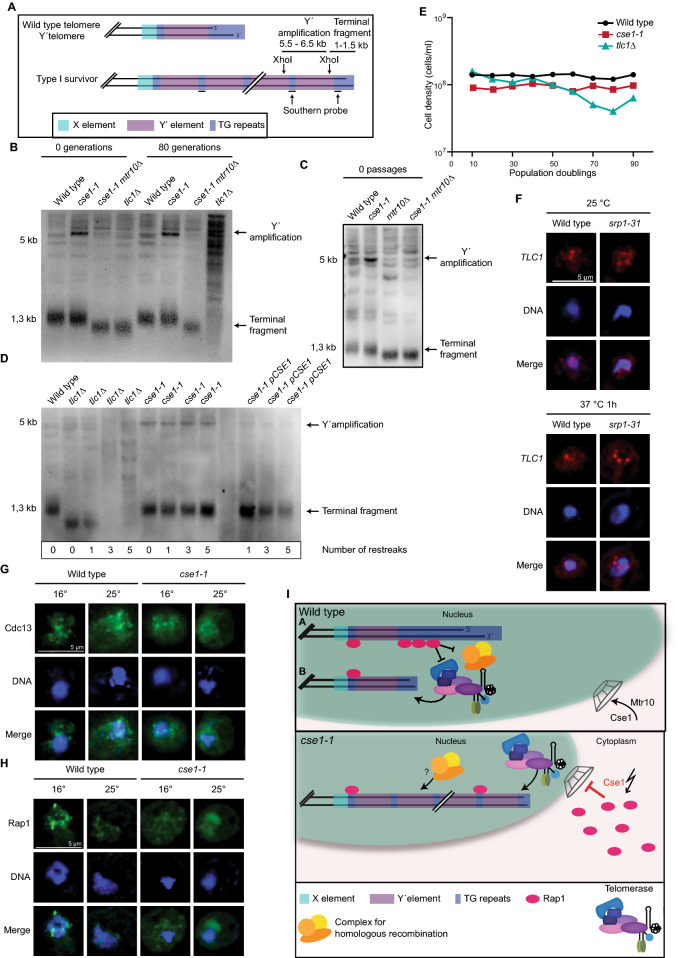
Mutation of *CSE1* results in Y′ element amplification. (**A**) Scheme of the Y′-amplification and location of the probe that was used for the southern blot. (**B**) The *cse1-1* mutation leads to the amplification of the telomeric Y′ element. XhoI digested genomic DNA of the indicated strains was used for the southern blot. The chromosome ends were detected with a digoxygenin labeled probe (**A**). Original image can be found in Supplementary Fig. S3A. (**C**) Mutation of *CSE1* leads to the amplification of of the telomeric Y′-element. Mutation of *MTR10* leads to shortend telomere ends. XhoI digested genomic DNA of the indicated strains was used for the southern blot. The chromosome ends were detected with a digoxygenin labeled probe (**A**). Image was cropped from the original. Original image can be found in Supplementary Fig. S3B. (**D**) Plasmid encoded *CSE1* recues the *cse1-1* phenotype. XhoI digested genomic DNA of the indicated strains was used for the southern blot. The chromosome ends were detected with a digoxygenin labeled probe (**A**). Original image can be found in Supplementary Fig. S3C. (**E**) Mutation of *CSE1* did not lead to a senescence phenotype. *n* = *1*. (**F**) *TLC1* localization is not altered in the *srp1-31* mutant. FISH experiments show no mis-localization of *TLC1* in *srp1-31* after shift to 37 °C for 1 h. *n* = *3*. (**G**) Cdc13 localization is not altered in the *cse1-1* mutant. Immunofluorescence experiments show no mis-localization of Cdc13 in *cse1-1* after shifting the cells to 16 °C for 1 h and 15 min. *n* = *3.* (**H**) Rap1 localization is altered in the *cse1-1* mutant. Immunofluorescence experiments shows nucleolar mis-localization of Rap1 in *cse1-1* at the permissive temperature and a cytoplasmic mis-localization after shifting the strain to 16 °C for 1 h and 15 min. *n* = *3*. (**I**) Model for Y′ element amplification in the *cse1-1* mutant. In wild type cells (upper panel) A, long and capped telomeres are usually not elongated via the telomerase. To short telomeres, B, telomerase is recruited and telomeres are elongated. In the *cse1-1* mutant (lower panel) defects in Rap1 import lead to unprotected telomeres, which triggers the Y′ amplification via homologous recombination.

Prevention of nuclear *TLC1* shuttling leads to shortened telomeres as seen in the export receptor Mex67 and Xpo1 as well as in the import receptor Mtr10^24,31^ (Fig. 3C). To investigate whether such defects would also be visible in the new import factor Cse1, we carried out southern blot analyses. Strikingly, we detected no shorter telomere ends in *cse1-1*, but instead highly amplified subtelomeric Y’ elements (Fig. 3A,B). Usually, cells that lack the telomerase or components of it undergo senescence when telomeres become critically short. The senescence phenotype is only temporary and most of these cells lose their viability, but a small percentage of cells overcome this phenotype by lengthening telomeres through homologous recombination, termed Type I and Type II survivors^40,41^. Type I survivors depend on Rad51 and exhibit highly amplified subtelomeric Y’ elements^42^, which are clearly visible in *cse1-1* cells (Fig. 3B,C). In contrast to the Type I survivors, *cse1-1* mutants have no shortened telomeres. It’s phenotype rather resembles a mutant in which telomere cap binding proteins, such as Rap1, are depleted.

In addition, we analyzed whether the telomeric phenotype in the *cse1-1* mutant can be reversed by introduction of a plasmid containing wildtype *CSE1*. Already after one restreak, which equals approximately 25 generations the Y' amplification band disapeared, indicating that the phenotype is based on the *cse1* mutation (Fig. 3D). Furthermore, it can be seen that upon re-streaking of the *tlc1∆* strain on a plate, Type I survivors are generated^43^ that have a short terminal fragment and Y′ amplification (Fig. 3D). This switch to homologues recombination in the *tlc1∆* goes along with a kink in the growth curve (Fig. 3E), which is absent in *cse1-1* cells.

Cse1 is also involved in the recycling of importin alpha, encoded by *SRP1* and *cse1-1* leads to an accumulation of Srp1 in the nucleus^33^. It was reported earlier that Srp1 might be involved in the nuclear localization of Est1^44^. Therefore, we examined whether *TLC1* mis-localizes in an *srp1-31* mutant. FISH experiments show no mis-localization of *TLC1* in *srp1-31* (Fig. 3F, Supplementary Fig. S2A,B).

Since short and uncapped telomeres are prone to recombination and often result in Y′ amplification we wondered whether an altered telomere cap structure in the *cse1-1* mutant might elicit the observed phenotype. Thus, we analyzed the localization of two important telomere cap components. While the localization of Cdc13 was not affected by the *cse1-1* mutation, Rap1 was mis-localized to the nucleolus at the permissive growth temperature of *cse1-1* and to the cytoplasm at the non-permissive temperature (Fig. 3G,H, ﻿Supplementary Fig. S2C,D). The resulting Rap1 depletion in the nucleus at telomeres might be the cause of the Y′ amplification in *cse1-1*.

### Pop protein loading onto *TLC1* occurs in the cytoplasm

**Figure 4:**
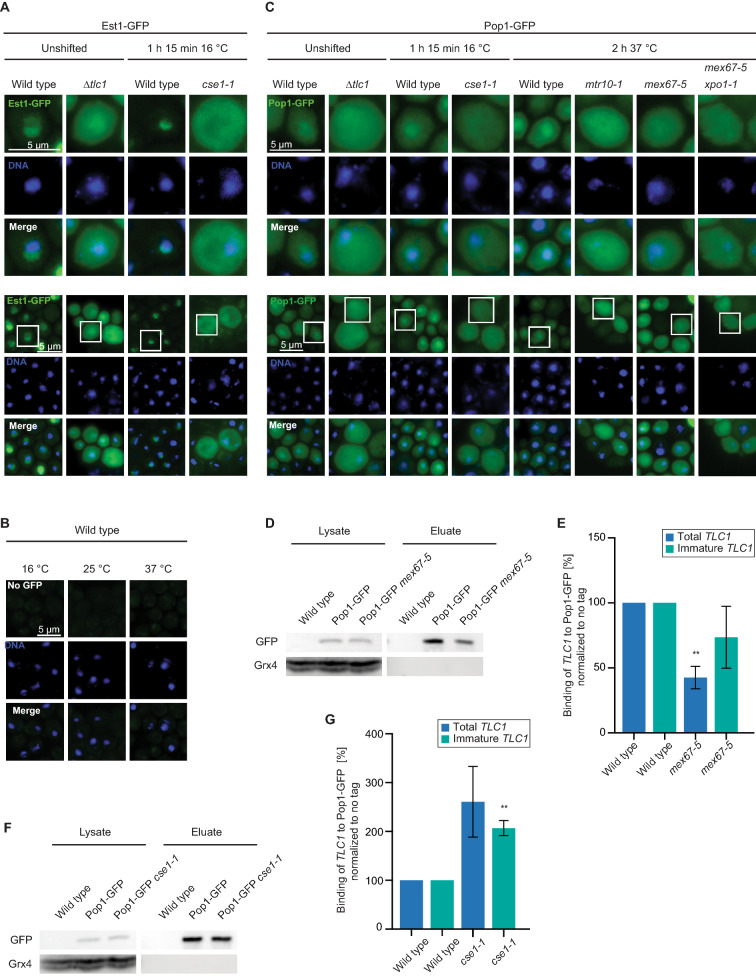
Loading of the Est and Pop proteins occurs in the cytoplasm. (**A**) Nuclear GFP-tagged Est1 mis-localizes to the cytoplasm in *cse1-1* cells. *n* = *3*. (**B**) A wild type strain is shown without a GFP tagged protein with identical microscopy settings as shown in (**A**). (**C**) Nuclear Pop1 localization is disturbed in mutants affecting *TLC1* localization. *n* = *3*. (**D**) Western blot of the Pop1-GFP IP is shown in the indicated strains. Grx4 served as a negative control. *n* = *3*. Image was cropped from the original. Original image can be found in Supplementary Fig. S4A. (**E**) Pop1 binding to *TLC1* is decreased in an export mutant. The Pop1-bound RNA from the several IP, one of which is shown in D, was analyzed in qPCRs *n* = *3*. (**F**) Western blot of the Pop1-GFP IP is shown in the indicated strains. Grx4 served as a negative control. *n* = *3*. Image was cropped from the original. Original image can be found in Supplementary Fig. S4A. (**G**) Pop1 binding to *TLC1* is increased in an import mutant. The Pop1-bound RNA from the several IP, one of which is shown in F, was analyzed in qPCRs. *n* = *3*.

After knowing the nuclear re-import requirements for *TLC1*, we asked, where the proteins of the RNP are loaded onto *TLC1* in the cell. We have suspected earlier that Est protein binding occurs in the cytoplasm, as the Est proteins accumulate in this compartment in a *tlc1∆* mutant^24^. Reassuringly, we also detect a cytoplasmic mis-localization for Est1-GFP in the new *TLC1* re-import factor mutant *cse1-1* (Fig. 4A,B).

For the Pop proteins the place of loading onto *TLC1* is currently unknown. As the proteins are mostly localized to the nucleolus and the nucleoplasm^45^, it is possible that they are loaded after *TLC1* has completed its cytoplasmic phase and returned to the nucleus. The function of the Pop proteins in *TLC1* maturation is to stabilize and recruit Est1 and Est2 within the holoenzyme^22,23^. Therefore, it is also conceivable that they are loaded to *TLC1* in the cytoplasm, before the Est proteins were properly attached. Importantly, Pop1 can only be stably associated with *TLC1* when the heterodimer Pop6 and Pop7 was attached to the RNA^46^. Thus, Pop1 binding is the final step in the loading of the Pop protein complex. We localized Pop1 in the *TLC1* nuclear import- and export mutants and in *tlc1*∆ and found a clear cytoplasmic mis-localization of the protein, even though we detect its localization in wild type not as strict nuclear as published earlier with the identical constructs^45^ (Fig. 4C). Interestingly, also mutations in the Pop proteins were shown to increase the cytoplasmic presence of *TLC1*^21^, which supports a model in which loading of the Pop proteins might occur in the cytoplasm. Thus, in situations, in which *TLC1* is not present in the cytoplasm, such as in *tlc1*∆ or in the export mutant *mex67-5 xpo1-1*, the Pop proteins accumulate in the cytoplasm and most likely do not bind *TLC1*. However, in the import mutants, one would expect that the proteins were loaded onto *TLC1*, but cannot be imported. To get additional support for a cytoplasmic loading and to find out whether Pop1 is loaded onto the long immature *TLC1* we carried out RIP experiments, with strains that contained endogenously tagged *POP1-GFP* expressed from its own promoter to avoid overexpression. Clearly, we found a decreased binding of this RNA in the export mutant (Fig. 4D,E), while an import block resulted in an increased binding of the immature *TLC1* to Pop1 (Fig. 4F,G), suggesting that the loading of the Pop proteins is indeed a cytoplasmic event.

### Mtr10 contacts *TLC1* at the Sm-ring for nuclear re-import

**Figure 5:**
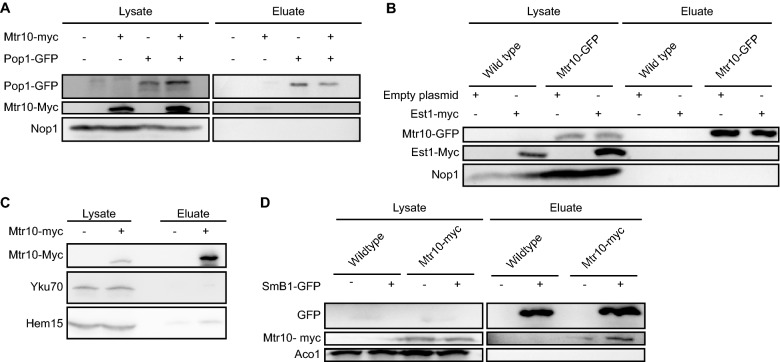
Both nuclear import receptors contact *TLC1* at the Sm-ring. (A) Mtr10 does not interact with the Pop protein complex. A western blot of co-IPs of GFP-tagged Pop1 with myc-tagged Mtr10 is shown. Nop1 served as a washing control *n* = *3.* Image was cropped from the original. Original image can be found in Supplementary Fig. S5.1A. (**B**) Mtr10 does not interact with the Est protein complex. A western blot of a co-IP of GFP-tagged Mtr10 with myc-tagged Est1 is shown. Nop1 served as negative control. *n* = *3.* Image was cropped from the original. Original image can be found in Supplementary Fig. S5.2A. (**C**) Mtr10 does not interact with the Yku protein complex. A western blot of a co-IP of myc-tagged Mtr10 with Yku70 is shown. *n* = *3.* Image was cropped from the original. Original image can be found in Supplementary Fig. S5.3A. (**D**) Mtr10 interacts with the Sm-ring. A western blot of a co-IP of GFP-tagged Smb1 with myc-tagged Mtr10 is shown. Aco1 served as a negative control. *n* = *3.* Image was cropped from the original. Original image can be found in Supplementary Fig. S5.3B.

Cse1 can mediate the nuclear import of snRNAs and *TLC1* only after the Sm-ring has properly assembled^25^ (Fig. 1E). An interaction between the Sm-ring and Cse1 has been shown before^25^. Their interaction thus resembles a quality control step to allow only nuclear entrance of the Sm-ring-bound *TLC1* RNP. It seems possible that also Mtr10 may contact *TLC1* only after proper protein loading has occurred. To analyze potential complex formations, we carried out co-immunoprecipitations (co-IPs) of Mtr10 with Pop1 and Est1. However, none of these proteins physically interacted with Mtr10 (Fig. 5A,B).

It is currently unclear whether the Yku-heterodimer is loaded in the cytoplasm. The Yku70-Yku80 heterodimer binds to a 48 nucleotides long stem loop of *TLC1* and is required for the nuclear localization of this RNA^19,29,47^ (Fig. 2A). Therefore, we investigated a potential binding between Mtr10 and Yku70 in co-IP experiments, but we did not detect an interaction (Fig. 5C).

Finally, we investigated, whether Mtr10 might, like Cse1, also interacts with the Sm-ring in co-IPs and indeed, a specific band of Mtr10 was detectable in the eluates of the Smb1-IPs (Fig. 5D). These findings indicate that both import receptors interact with the Sm-ring to support nuclear import of the matured *TLC1* RNP.

### Processing of *TLC1* occurs in the nucleus after re-import of the protein bound RNP

The longer pre-*TLC1* is trimmed by the exosome up to the Sm-ring, resulting in a 1158 nucleotides long form^7^. Our studies in the re-import mutants suggest that trimming occurs after shuttling, because of an increased Smb1-binding to the immature precursor RNA of *TLC1* (Figs. 1, 2H). Moreover, mutants that accumulate *TLC1* in the cytoplasm accumulate immature *TLC1* at decreasing total *TLC1* levels (Fig. 2I–K), suggesting that the *TLC1* RNA is not trimmed before its journey through the cytoplasm.

**Figure 6:**
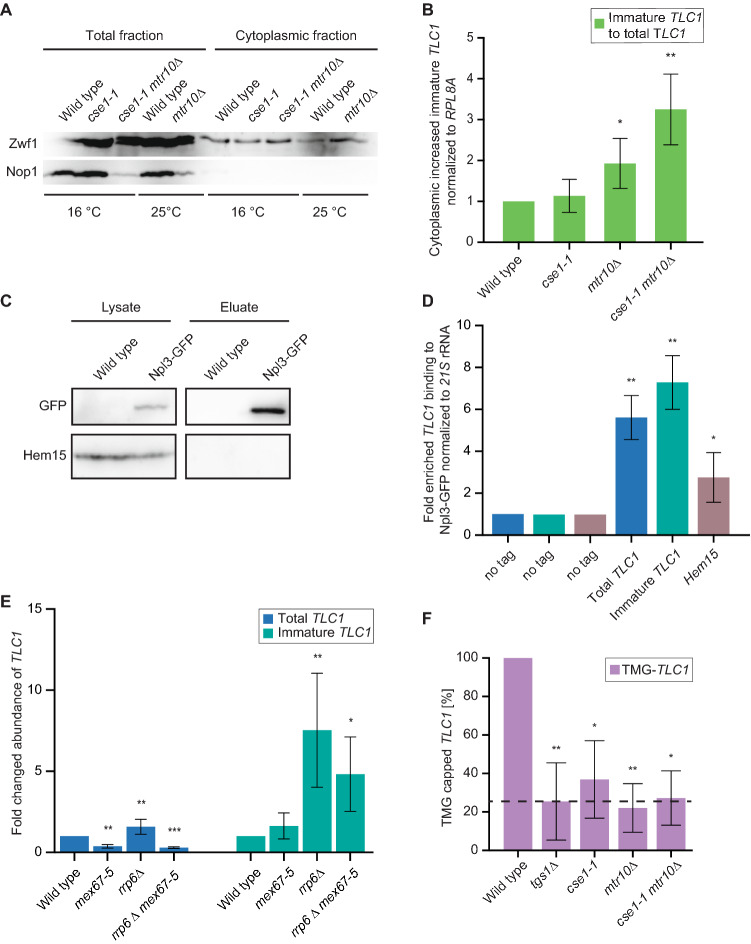
Processing and TMG-capping of *TLC1* occurs in the nucleus upon *TLC1* re-import. (**A**) Immature *TLC1* accumulates in import factor mutants. A Western blot of a nucleo-cytoplasmic fractionation experiment is shown for the indicated strains. The nuclear Nop1 protein and the cytoplasmic Zwf1 protein served as controls for successful isolation of the cytoplasmic fraction. Strains were shifted to 16 °C for 1 h 15 min prior to the experiments. *n* = *5.* Image was cropped from the original. Original image can be found in Supplementary Fig. S6A. (**B**) qPCRs of the fractionation experiments shown in (**A**) reveals an accumulation of the immature *TLC1* in the import factor mutants at the permissive termperature. *n* = *5.* (**C**) Western blot showing an example of a Npl3 IP used in (**D**) in the RIP experiments. Image was cropped from the original. Original image can be found in Supplementary Fig. S6B. (**D**) *TLC1* binds to Npl3. The Npl3 co-precipitated RNA was used as a template in qPCRs for *TLC1* and Hem15 as positive control. *n* = *4.* (**E**) Trimming of *TLC1* is mediated by the nuclear exosome after nuclear re-import. The RNA was isolated from the indicated strains after shifting them to 37 °C for 2 h. Subsequently, qPCRs were carried out that determined the amount of the total *TLC1* and its immature, longer precursor. *Mex67-5 and rrp6∆ mex67-5 n* = *4. Rrp6∆ n* = *8.* (**F**) TMG-capping occurs after nuclear *TLC1* re-import. The amount of TMG-capped *TLC1* was determined by TMG-co-IPs and subsequent qPCRs from the indicated strains. *tgs1∆* served as a positive control and the black line indicates the amount of the precipitated non-TMG-capped RNAs resembling the base line for TMG-capping. *Tgs1∆* and *mtr10∆ n* = *4. Cse1-1* and *cse1-1 mtr10∆ n* = *3.*

To further analyze this point, cytoplasmic fractionation experiments were carried out. Compared to wild type cells, more immature than total *TLC1* accumulates in the cytoplasm of the import factor mutants (Fig. 6A,B). These findings suggest that the immature form shuttles into the cytoplasm and trimming occurs afterwards by the nuclear exosome. Secondly, we assayed the *TLC1-*forms in the *mex67-5* nuclear export mutant. We detected a decrease of total *TLC1*, which is in agreement with earlier findings in which Mex67 was suggested to protect *TLC1* from its degradation^30^. However, in our experiments we still see a two-fold increase of the immature *TLC1* in *mex67-5* mutants, which suggests that immature *TLC1* is not immediately degraded when Mex67 is missing (Fig. 6E). Possibly, other proteins such as the guard proteins, one of which is Npl3, that recruit Mex67 protect the immature *TLC1* from being degraded while waiting for export. To test whether Npl3 binds *TLC1*, we precipitated the protein and found *TLC1*, in particular the immature form to bind Npl3 (Fig. 6C,D). To address whether the trimming to the mature *TLC1* occurs via the nuclear exosome, we analyzed *TLC1* in *rrp6∆*. Its deletion leads to an approximately two-fold increase of the total *TLC1*, which includes the mature form and the immature form, and a ~ sevenfold increase of the immature form of *TLC1* (Fig. 6E), indicating that the immature form is trimmed in the nucleus. Remarkably, although the RNA is less stable in *mex67-5* mutants, the immature *TLC1* still increased five-fold in the double mutant *rrp6∆ mex67-5*, suggesting (a) that its trimming occurs in the nucleus and (b) that the immature form is still protected without Mex67. Furthermore it has to be noted, that in contrast to Vasianovich and colleagues, we find no rescue of the decreased *TLC1* in the double mutant *rrp6∆ mex67-5* as detected by Northern blot^30^. Since in our experiments we applied the highly sensitive qPCR method, we were able to detect an increase of the immature *TLC1* when its export is blocked in *mex67-5*, despite the overall decrease of the total *TLC1* in both mutants (Fig. 6E).

Taken together, these finding show that trimming of the immature *TLC1* requires nuclear export and that correct trimming occurs only after Sm-ring loading, which seems logic, because the Sm-ring was shown to protect *TLC1* from full degradation^17^. Our data furthermore suggest that the immature *TLC1* is protected from degradation by Mex67 binding and its immediate export activity into the cytoplasm. But they also suggest that additional proteins, like Npl3, protect the immature *TLC1* from degradation, possibly the guard proteins that recruit Mex67 after quality control, as the immature form increases in the nucleus of *mex67-5* mutants (Fig. 6D,E).

Another modification of *TLC1* is the TMG-capping at its 5’-end in the nucleolus by Tgs1^11,48^. To investigate, whether this trimethylation occurs before or after shuttling we carried out RIPs experiments with a TMG-cap specific antibody. Subsequently, we carried out qPCRs to detect *TLC1*. Compared to wild type, we found an approximately 30% decrease in the level of the TMG-capped *TLC1* in a *TGS1* deletion strain, which resembles the zero-line for the unspecific binding of this antibody, because Tgs1 is the only trimethyltransferase in yeast. Clearly, also in the single import mutants *mtr10*∆ and *cse1-1* as well as in the double mutant we found a reduction, which was around 30% (Fig. 6F). These findings indicate that TMG-capping occurs after shuttling.

**Figure 7:**
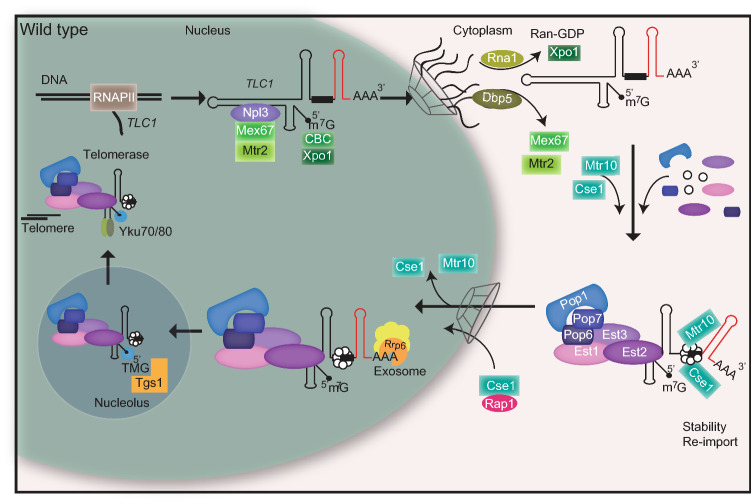
Model for the compartmental stepwise maturation of *TLC1*. The longer precursor of *TLC1* is transcribed in the nucleus and immediately exported into the cytoplasm upon binding of the export receptor Mex67-Mtr2 and the karyopherin Xpo1/Crm1, the latter of which interacts with CBC bound m^7^G-caps. Upon export, Mex67 and Xpo1/Crm1 are displaced and the Sm-ring, the Est and the Pop proteins assemble on the immature *TLC1* in the cytoplasm. Subsequently, this RNP is re-imported back into the nucleus via Mtr10 and Cse1, both of which contact *TLC1* via its Sm-ring. Thus, nuclear re-import can only occur after RNP formation including the Sm-ring and therefore resembles an important quality control checkpoint. In the nucleus, the import receptors dissociate and *TLC1* is trimmed up to the Sm-ring by the nuclear exosome. Finally TMG-capping occurs in the nucleolus assisted by Smb1, which interacts with Tgs1. This step terminates shuttling, because export receptors cannot be loaded anymore. The matured holoenzyme subsequently acts in telomere maintenance.

Taken together, we have uncovered a stepwise maturation cycle for the telomerase, which is summarized in Fig. 7. Maturation begins with the nuclear export of the longer pre-*TLC1* into the cytoplasm by Mex67, probably assisted by Npl3 and Xpo1 that binds to the CBC. In the cytoplasm not only the Sm-ring is loaded, but also the Est- and the Pop proteins attach to the immature *TLC1*. Its successful assembly allows Mtr10 and Cse1 to bind and re-import pre-*TLC1* into the nucleus. Subsequently, trimming of the immature form into the mature 1158 nucleotides long form is mediated by the nuclear exosome and TMG capping finally occurs in the nucleolus by Tgs1.

In our compiled investigation we have ordered the maturation process of *TLC1* and show the order of events from nuclear export of the premature transcript to the binding of the Est- and Pop proteins and the Sm-ring in the cytoplasm to its trimming and TMG-capping after re-import into the nucleus. We show that the Sm-ring binding resembles a quality control step that enables subsequent movement of the RNP into the nucleus. Thus, the step-wise maturation of *TLC1* is controlled by its compartmental shuttling.

## Discussion

Functional telomerases are important to prevent the repetitive shortening of the DNA ends after each round of replication for recurrent cell divisions. Due to the fact that these holoenzymes undergo a stepwise assembly, a danger is that incompletely assembled telomerases interfere with the process of telomere elongation. In the worst case, these immature telomerases could, due to their restricted or absent functionality, prevent telomere maintenance. To avoid such a scenario, a mechanism has developed, in which the RNA scaffold, *TLC1* in yeast, is exported into the cytoplasm via the export receptors Mex67-Mtr2 and Xpo1/Crm1^24,29^. While Xpo1/Crm1 contacts the CBC, which is bound to the monomethyl cap of the RNA-polymerase II transcribed RNA, Mex67-Mtr2 was speculated to contact the RNA via the adapter proteins Npl3, Gbp2 and Hrb1, because such contact is observed for mRNA and snRNA export^25–28^.

Binding of the Est proteins to *TLC1* and association of the Sm-ring were suggested to be cytoplasmic events, while TMG-capping and trimming of *TLC1* were suggested to occur in the nucleus prior to shuttling^24,29,30^. Up to date, the place of the Pop protein loading onto *TLC1* was unknown, but suspected to occur in the cytoplasm, because mutants of *POP1* and *POP6* accumulated *TLC1* in the cytoplasm^21^. We collected additional evidence for a cytoplasmic Pop protein loading and analyzed the localization of Pop1 in the import factor mutants. We found a cytoplasmic accumulation of Pop1 in the mutants (Fig. 4C), supporting the idea of their cytoplasmic loading and supporting the newly discovered role of Cse1 in *TLC1* nuclear import. Finally, we could confirm this result through RIP experiments in which we detected a decreased *TLC1* binding to Pop1 in *mex67-5* and an increased binding in the import mutant *cse1-1* (Fig. 4E,G). As the function of the Pop proteins is to stabilize the Est proteins on the RNA and to support the RNP structure^22,23^ their cytoplasmic loading seems to be a final step before re-import.

Another important event is the loading of the Sm-ring that limits the trimming of the *TLC1* precursor, which was recently shown to occur in the cytoplasm, similar to the Sm-ring loading onto snRNAs^25,30^. However, in the interesting study of Vasianovich and colleagues a modified *TLC1* that contained several MS2 stem loops was used, which could have an influence on its localization. In addition, they could not distinguish which form of *TLC1* is accumulating in the cytoplasm. In our study we used probes and primers that (1) detect the unmodified natural *TLC1* and (2) selectively detect the immature pre-*TLC1*. With this method we confirmed the data of Vasianoivich and colleagues and show that the Sm-ring loading is a cytoplasmic event. But in addition, we have shown that the Sm-ring and the Pop proteins associate with the immature precursor of *TLC1* in the cytoplasm (Figs. 1I, 2H, 4G) and that trimming occurs after Sm-ring loading and after re-import into the nucleus (Figs. 1I,K, 2H,K, 4E,G, 6E).

It was shown that the karyopherin Cse1 contacts the Sm-ring and particularly Smb1 for nuclear re-import of the snRNAs^25^. Interestingly, we also detected a binding between the Sm-ring and the other import factor, Mtr10 (Fig. 5D). This finding is important for three reasons. First, besides Mtr10, Cse1 is another import factor that facilitates the transit of this huge RNP through the NPC. The more transport factors are involved in transporting large particles, the better the transit is working. This is supported by the additive effect for the mis-localization of *TLC1* seen in the combined import factor mutants (Fig. 6A,B). Secondly, a potential failure of one of the import receptors does not fully prevent *TLC1* re-import. Third, nuclear re-import supported by Mtr10 and Cse1 can only occur on *TLC1* molecules that have received the Sm-ring, which resembles an elegant way of cellular quality control for this important maturation step of the telomerase. For this, Cse1 might be more important because we detect a decreased binding of *TLC1* to Smb1 in *cse1-1* cells, even though we see no drastic change in the overall amount of *TLC1* (Fig. 2F–K). Together, these findings argue for a control step in the RNP formation, which prevents that faulty RNPs can enter the nucleus and jeopardize telomere function. However, we cannot exclude that another protein is involved in its stabilization, which is dependent on Cse1 transport. In every sense, Sm-ring loading is an essential step in maturation and allows nuclear re-import of pre-*TLC1*.

Interfering with the shuttling of *TLC1* either by mutation of the export factors Mex67 and Xpo1 or the import receptor Mtr10 was shown to result in telomere shortening defects^24,31^. Interestingly, we did not detect any telomere shortening defects and no senescence phenotype for the novel *TLC1* re-import factor mutant *cse1-1* (Fig. 3B,E). Instead, mutation of *CSE1* resulted in an Y′ element amplification (Fig. 3A,B). In addition to a functional telomerase an altered telomere cap structure can also impact the telomere length homeostasis. Cap-protecting proteins are the dsDNA binding protein Rap1 and the ssDNA binding protein Cdc13. Since Rap1 is either interacting with the Sir complex on centromere proximal regions or with the Rif1 and Rif2 proteins on centromere distal regions, one might speculate that either the nuclear import of Rap1 itself, or one of its tethering proteins might depend on Cse1 for nuclear import. Indeed, we found a cytoplasmic mis-localization of Rap1 in *cse1-1* that supports this idea. Interestingly, Rap1 was also mis-localized at the permissive temperature of *cse1-1*. However, in contrast to the non-permissive temperature, it was found in the nucleolus, not in the cytoplasm, which might also suggest that its telomere tethering factors were absent, possibly not properly imported by Cse1.

Even though Cse1 mediates the re-import of *TLC1*, mutation of this import factor additionally impacts proper telomere cap formation, which might elicit the observed phenotype. Here, the importer seems to have a function in transporting Rap1 or a factor that prevents proper Rap1 localization on chromosome ends.

Furthermore, one can speculate that either Rad51 itself, or factors of the Rad51 homologous recombination pathway might not be imported in *mtr10*∆, so that *mtr10∆* mutants cannot use homologous recombination for telomere maintenance but rather show a shortening of the telomeres.

Moreover, we have excluded an involvement of Srp1 in the localization of *TLC1*. Srp1 was suggested to mediate the nuclear localization of Est1 and since Srp1 mis-localizes in the *cse1-1* mutant an influence also on *TLC1* was conceivable^33,44^. However, *TLC1* and thus telomerase localization does not appear to be dependent on Srp1, as mutation of *SRP1* does not lead to a mis-localization of *TLC1* (Fig. 3F).

At steady state about 90% of *TLC1* are present in the mature shorter form which is present in the functional telomerase and only a small fraction is present as the polyadenylated longer form^9,13^. Older model discuss the CPF-CF terminated poly(A)^+^ form as a precursor of the mature form^13^, but more recent studies suggest that the NNS terminated form might be the prominent one^15^. Therefore, it is currently unclear how much of the *TLC1* originates from NNS or CPF-CF termination. However, since both versions (a) need to adopt the Sm-ring for general protection, which was shown to be a cytoplasmic process^25^ and (b) total *TLC1* significantly decreases if shuttling is prevented (Fig. 2I) and (c) leads to defects of the telomeric structure (Fig. 3) and Ref.^24^, shuttling of any immature form seems highly relevant.

It was unclear when trimming occurs. Earlier studies indicated that mutations in the nuclear component of the exosome resulted in a strong increase of *TLC1*^17^. However, the experimental set up did not allow to distinguish whether the exosomal trimming occurs before or after shuttling. To determine the time and place of the *TLC1* trimming, we first showed that the immature form accumulates in nuclear re-import mutants and in mutants that are defective in the Sm-ring assembly (Figs. 1 and 2I–K). Additionally, we have shown by nucleo-cytoplasmic fractionation experiments that the immature form accumulates in cytoplasm in the import factor mutants (Fig. 6B), which indicates that trimming occurs after re-import. Furthermore, mutation of *RRP6* resulted in a strong increase of the immature pre-*TLC1* (Fig. 6E), suggesting that the nuclear exosome is responsible for trimming after shuttling. This finding also suggests that the immature form is still protected without Mex67. One possibility is that the Mex67-interacting and RNA-binding proteins Npl3, Gbp2 and Hrp1 might guard *TLC1*. And indeed, we have shown that *TLC1* and especially the immature *TLC1* is bound by Npl3 (Fig. 6D). Co-transcriptionally loading of Npl3 prior to binding of Mex67^49,50^ provides coverage and thus protection of the RNP.

The finding, that Sm-ring binding occurs before trimming seems logical, because unprotected RNA is an ideal substrate for the exosome^18^. Interestingly, preventing nuclear export by mutation of *MEX67* leads to a decrease of *TLC1* (Fig. 6E and Ref.^30^). During the short period of time in which the immature *TLC1* RNA is usually present in the nucleus before Mex67 and Xpo1 export it into the cytoplasm, the nuclear exosome is not able to attack the RNA sustainably. Thus, Mex67 cannot be the only protecting RNA-binding protein, because even in mutants of this export receptor we detect a ~ twofold and a ~ fivefold increase of the immature *TLC1* in *mex67-5* and *rrp6∆ mex67-5*, respectively, as compared to wild type. Possibly this is achieved by guard proteins such as Npl3 that recruit Mex67^28,50^.

Our finding that the Sm-ring loading occurs in the cytoplasm prior to TMG-capping is in agreement with other studies that have shown that the Sm-ring is crucial for trimethylation of the cap^51^. Furthermore, we have shown earlier that snRNAs are retained in the nucleolus when Smb1 is depleted that showed that an intact Sm-ring is required for efficient trimethylation and subsequent nucleolar release into the nucleoplasm^25^. It is well conceivable that the formed Sm-ring assists TMG capping, as it interacts with Tgs1 both, in vivo and in vitro^11,25^.

Importantly, snRNA nucleo-cytoplasmic shuttling was suggested to be terminated by trimethylation of the 5’ cap^25^. The export receptor Xpo1 interacts with the 5’ cap binding complex CBC, in particular with Cbp80^25^. This might be similar for *TLC1*, because pre-*TLC1* contains an m^7^G cap, which is bound to CBC (Fig. 1K) and *TLC1* interacts with Xpo1^24^. After receiving the TMG cap, CBC might not be bound and Xpo1 then unable to interact. Since the other export receptor, Mex67, is already displaced at the NPC when entering the cytoplasm^52,53^, Xpo1 is the only transport factor left for a repeated snRNA export and TMG capping could close this option. Therefore, it seems logic that TMG capping on *TLC1* also occurs after shuttling and maturation and we could indeed show that preventing nuclear re-import of *TLC1* results in the accumulation of m^7^G, but not TMG capped RNAs (Fig. 6F).

Together, our analysis uncovered the steps in which telomerase maturation takes place (Fig. 7) and identified that proper cytoplasmic assembly of the telomerase is prerequisite for nuclear re-import and thus represents a quality control check point in the life cycle of this holoenzyme. Any way of interfering with the compartmental maturation process inevitably leads to defects in telomere biology (Fig. 3) and^19,21,24,29,31,54,55^, reflecting the importance of this step-wise process.

## Experimental procedures

### Yeast strains, plasmids and oligonucleotides

All yeast strains used in this study are listed in the Supplementary Table S1, oligonucleotides in ﻿Supplementary Table S2 and plasmids in ﻿Supplementary Table S3. Plasmids and yeast strains were generated by conventional methods.

### Fluorescent in situ hybridization experiments (FISH)

The experiments were essentially carried out as described^25^. RNA probes were with Cy3-labeled oligonucleotides (Sigma), which are listed in Supplementary Table S3. Cells were grown to mid log phase (1 × 10^7^ cells/ml) prior to temperature shift to 37 °C 1 h or 2 h or to 16 °C for 1 h 15 min (Figs. 2A, 3F, Supplemantry Fig. S2A–C, Supplemantry Fig. S3D,E). For Sm-ring dependent localization studies, cells were grown to log phase in YP medium containing 2% galactose. Afterwards 4% glucose was added and cells were incubated at 25 °C for 2 h (Fig. 1E). Samples were fixed by adding formaldehyde to a final concentration of 4% for 45 min at room temperature. Cells were spheroplasted by adding zymolyase, subsequently permeabilized in 0.1 M potassium phosphate buffer pH 6.5, 1.2 M sorbitol, 0.5% Triton® X-100, pre-hybridized with Hybmix (50% deionized formamide, 5 × SSC, 1 × Denhardts, 500 μg/ml tRNA, 500 μg/ml salmon sperm DNA, 50 μg/ml heparin, 2.5 mM EDTA pH 8.0, 0.1% Tween® 20) for 1 h on a poly-l-ysine coated slide at 37 °C and hybridized in Hybmix with the specific probe over night at 37 °C. After hybridization, cells were washed with 2 × SSC and 1 × SSC at room temperature, each for 1 h and 0.5 × SSC at 37 °C and room temperature, each for 30 min. DNA was stained with Hoechst 33342 (Sigma). Microscopy studies were performed with a Leica AF6000 microscope and pictures were obtained by using the LEICA DFC360FX camera and the LAS AF 2.7.3.9 software (Leica). For deconvolution (Fig. 1E, 2A, 3F, Supplemantry Fig. S2A–C, Supplemantry Fig. S3D,E) z-stacks (10 stacks; 0.2 µm) were recorded and the maximal projection was deconvoluted with 3 iterations by the LAS AF 2.7.3.9 software (Leica).

### Immunofluorescence experiments

The experiments were essentially carried out as described in^24^. For signal enhancement of GFP-tagged proteins, they were visualized using a primary anti-GFP and a secondary FITC-coupled antibody. Cells were grown to mid log phase (1 × 10^7^ cells/ml) prior to a temperature shift to 16 °C for 1 h 15 min (Fig. 3G,H, Supplemantry Fig. S3F,G). Cells were fixed in a final concentration of 4% formaldehyde for 10 min at the restrictive temperature and a 50 min incubation at 25 °C. Cells were spheroblasted using zymolyase and placed on a poly-l-lysine coated slide. The cells were permeabilized with 0.1 M potassium phosphate buffer pH 6.5, 1.2 M sorbitol, 0.5% Triton® X-100. Afterwards they were blocked for 1 h in antibody blocking buffer (0.1 M Tris pH 9.0, 0.2 M NaCl, 5% heat-inactivated FCS and 0.3% tween). The buffer was removed and replaced with a 1:1.000 dilution of the primary anti-GFP (Thermo Fisher) mouse antibody in antibody blocking buffer and incubated for 2 h at room temperature. The cells were washed twice shortly with 1 × PBS, 0.5% tween and once for 30 min. Afterwards the cells were washed once with 1 × PBS and once with antibody blocking buffer each for 30 min. The secondary aqntibody (anti-mouse IgG-FITC; Sigma) was diluted 1:100 in antibody blocking buffer and incubated for 1 h at room temperature. Finally, the cells were washed once quickly with 1 × PBS, 0.5% tween and once for 30 min. Subsequently, cells were washed twice with 1 × PBS for 15 min each. DNA staining and microscopy was performed as described in the FISH experiment.

### RNA co-immunoprecipitation experiments (RIP)

All yeast strains were grown to mid log phase (2 × 10^7^ cells/ml). For RIP experiments seen in Fig. 1A,B,J,K, 2C,D and 6C,D (Supplemantry Fig. S1A,D, 2D, 6B) cells were cultured at 25 °C, in 1C, D, G-I; 2E-H; 4D-G and 6F (Supplemantry Fig. S1B,C, 2E, 4A) cells were shifted to a non-permissive temperature for 1 h 37 °C or 1 h and 15 min 16 °C respectively. Afterwards cells were harvested and lysed in RIP buffer (25 mM Tris HCl pH 7.5, 100 mM KCl, 0.2% (v/v) Triton X-100 (1% Triton X-100 for 2E), 0.2 mM PMSF, 5 mM DTT, 10 U RiboLock RNase Inhibitor (Thermo Scientific) and protease inhibitor (Roche) using the FastPrep®-24 machine (MP Biomedicals) three times for 30 s at 5.5 m/s. After centrifugation the supernatant was incubated for 1 h at 4 °C with GFP-Selector beads (NanoTag) (Fig. 1B,D,H,I,K, 2D,F–H, 4E,G and 6D). For TMG-cap-IPs total RNA was extracted from yeast lysates using trizol-chloroform (Ambion® RNA by Life technologies™). 50 µg of the total RNA was incubated for 1 h at 4 °C with 10 µl of the anti-2,2,7-trimethylguanosine-antibody (Calbiochem Milipore) coupled to sepharose beads (Fig. 6F).

The beads were washed five times with RIP buffer and for GFP-RIP split in two portions after the last washing step. Proteins were detected by western blot (1A, C, G and J; 2C, E; 4D, F and 6C). Eluates were purified via trizol-chloroform (Ambion® RNA by Life technologies™) extraction. The purified RNA was measured via Nanodrop and a defined amount of RNA was reverse transcribed with FastGene Scriptase II (Nippon Genetics) for subsequent qPCR analyses. All eluates were related in each strain to the lysates and substracted from the no-tag controls to account for strain-specific variations in the total amount. To normalize the total RNA amount, we used the mitochondrial *21S* rRNA. In Figs. 2H and 6B, the enrichment of the unprocessed form was shown in comparison to total *TLC1* amount.

### Total RNA isolation

Total RNA Isolation was carried out with the NucleoSpin RNA Kit from Macherey–Nagel (Figs. 2I–K, 6E). All steps were performed according to the manufactures description, except step 7. The DNA digestion on the column was executed for 1 h. An additional DNA digestion step was carried out after elution of the RNA. For the additional DNA digest the eluted RNA was mixed with a 10th volume of the Reaction Buffer for rDNAse and 1 µl rDNAse, according to the manufactures description. The digest was incubated for 1 h at 37 °C, before it was terminated through sodium acetate ethanol precipitation. For this 0.1 volume 3 M sodium acetate, pH 5.2, 2.5 volumes of 99% pure ethanol and 1 µl glycoblue were mixed with the RNA and incubated over night at − 20 °C. The purified RNA was measured via Nanodrop and a defined amount of RNA was reverse transcribed with FastGene Scriptase II (Nippon Genetics) for subsequent qPCR analyses.

### Nucleo-cytoplasmic fractionation experiment

For the detection of *TLC1* in the cytoplasm (Fig. 6B, Supplementary Fig. S6A) cells were grown to mid log-phase (2 × 10^7^ cells/ml). The cells were harvestet by centrifugation for 5 min at 4000 rpm. Cells were washed once with 1 ml YPD/ 1 M Sorbitol/ 2 mM DTT and resuspended in YPD/ 1 M Sorbitol/ 1 mM DTT. Cells were spheroblasted using 1 mg zymolyase (100 mg/ml) and after that diluted in 50 mL YPD/ 1 M Sorbitol for 30 min at 25 °C for recovery. Subsequently, the cells were shifted to 16 °C for 1 h 15 min (*cse1-1* and *cse1-1 mtr10∆)*. Cells were placed on ice, centrifuged at 2000 rpm for 10 min and the pelleted cells were resuspended in 500 μl Ficoll buffer (18% Ficoll 400, 10 mM HEPES pH 6.0) and 1 µl Ribolock. Cells were lysed by addition of 1 ml buffer A (50 mM NaCl, 1 mM MgCl_2_, 10 mM HEPES pH 6.0). The suspension was mixed and centrifuged at 4000 rpm for 15 min. The supernatant was used for cytoplasmic analyses. RNA was isolated using the Nucleo-Spin RNA Kit (Macherey and Nagel). The purified RNA was reverse transcribed with FastGene Scriptase II (Nippon Genetics) for subsequent qPCR analyses. All values were normalized to the amount of the mRNA present for *RPL8A*. To verify no nuclear contamination in the cytoplasmic fraction, aliquots of the samples were analyzed in Western blots for the presence of the cytoplasmic Zwf1 protein and the absence of the nuclear Nop1 protein (Fig. 6A).

### GFP-microscopy

Cells were grown, treated and harvested as described in the FISH experiments. Cells were fixed with 3% formaldehyde for 2 min at room temperature, subsequently washed with 0.1 M phosphate buffer pH 6.5 and with P-solution (0.1 M phosphate buffer pH 6.5, 1.2 M Sorbitol), before an aliquot was added to a poly-l-lysine-coated slide for 30 min at 4 °C. Permeabilization of the cells, DNA staining and microscopy was performed as described in the FISH experiment.

### Co-immunoprecipitation (IP) experiments

All yeast strains were grown to log phase (2–3 × 10^7^ cells/ml). Afterwards, the cells were harvested and lysed in IP buffer (1 × PBS, 3 mM KCl, 2.5 mM MgCl_2_, 0,5% Triton X-100 and protease inhibitors from Roche). 35 µl of this lysate was loaded onto an SDS-gel (lysate lanes). The supernatant was incubated for 1 h at 4 °C with GFP-Selector beads (NanoTag) (Fig. 5A,B,D, Supplementary Fig. S5.1A, 5.2A, 5.3B) or with Myc-trap beads (Chromotek) (Fig. 5C, Supplementary Fig. S5.3A). The beads were washed five times with IP buffer, and finally resuspended in 35 µl SDS-sample buffer. The entire eluate sample was loaded onto the SDS-gels.

Subsequently, the proteins were detected by Western blot analyses with the indicated antibodies (GFP (Chromotek) 1:4,000; c-myc (9E10) (Santa Cruz) 1:1,000; Hem15 and Grx4 each 1:5,000 and Aco1 1:2,000 (U. Mühlenhoff); Nop1 (Santa Cruz) 1:4,000; Hdf1 (Yku70) (Santa Cruz) 1:4,000). Signals were detected with the Fusion SL system (PeqLab) and FusionFX7 Edge (Fusion FX Vilber). To be able to detect several proteins in one experiment, the western blots were cut horizontally according to the size of the desired proteins to be able to detect each stripe with individual antibodies.

### Drop dilution analysis

Cells were grown to log phase (2–3 × 10^7^ cells/ml) and diluted to 1 × 10^7^ cells/ml. Tenfold serial dilutions to 1 × 10^3^ cells/ml were prepared and 10 µl of each dilution was spotted onto full medium (YPD) agar plates that were subsequently incubated for 3 days at the indicated temperatures (Fig. 2B). Pictures were taken after 2 and 3 days with the Intelli Scan 1600 (Quanto technology) and the SilverFast Ai program.

### Southern blot analysis

Yeast strains were either passaged in liquid culture (Fig. 3B,C, Supplementary Fig. S3A,B) or on solid agar plates (Fig. 3D, Supplementary Fig. S3C). For passaging in liquid culture, cells were inoculated with a starting concentration of 1 × 10^5^ cells/ml. The strains were grown for 3 days per passage at 20 °C until they reached 1 × 10^8^ cells/ml and were afterwards diluted again to 1 × 10^5^. Overall 8 passages, corresponding to 80 generations, were carried out. Cell passages on solid agar plates were used for generating Type I survivors in *tlc1∆* cells. The strains were freshly restreaked form the database and single colonies were restreaked after 2–3 days at 25 °C. One restreak comprises about 25 generations. Overall 5 restreaks were generated coresponding to about 125 generations.

Genomic DNA was isolated with the MasterPure Yeast DNA Purification Kit (Lucigen). This gDNA was digested with XhoI (Nippon Genetics) according to the manufactures description. 20 µg gDNA were loaded per lane and electrophoretically separated on a 1% TBE gel at 4 °C for 3 h. The gel was depurinated (250 mM HCl) for 15 min, denatured (1.5 M NaCl, 0.5 M NaOH) for 30 min, neutralized (1.5 M NaCl, 0.5 M Tris–HCL, pH 7.5) for 30 min and equilibrated in 20 × SSC (0.3 M Tri-sodium citrate, 3 M NaCl, pH 7.0) for 15 min. A capillary blot was used for the transfer onto a HybondN^+^ (GE Healthcare) membrane. After transfer, the membrane was crosslinked for 7 min by UV light (254 nm, 120,000 µJ/cm^2^) and then heated to 80 °C for 2 h. Subsequently, pre-hybridization was carried out for 1 h at 68 °C in pre-hybridization buffer (0.5 M sodium phosphate buffer pH 7,5; 7% (w/v) SDS; 1 mM EDTA). For hybridization, a digoxygenin-labeled probe, detecting the TG repeats of the telomeres, was added to the pre-hybridization solution and incubated over night at 37 °C with rotation. After washing, the southern blot was detected by using an anti-digoxygenin antibody coupled to alkaline phosphatase (Roche) and the labeling and detection starter kit (Roche).

### Senescence assay

Yeast strains were passaged every 10 generations in fresh liquid medium (Fig. 3E). Strains were grown at the semi-permissive temperature of 20 °C.

### Quantification

All experiments shown in this work were performed in biologically independent repetitions as indicated in the figure legends. Error bars represent the standard deviation. P values were calculated using a one or two-tailed, two-sample unequal variance t-test. P values are indicated as follows: ***p < 0.001, **p < 0.01, *p < 0.05.

## Supplementary Information

Supplementary Information.

## Data Availability

The datasets generated and/or analyzed for this study are available from the corresponding author upon reasonable request.
