# Supplementary material for: Unraveling the stepwise maturation of the yeast telomerase including a Cse1 and Mtr10 mediated quality control checkpoint
Source: Sci Rep. 2021 Nov 12;11:22174. doi: 10.1038/s41598-021-01599-3 (PMC8590012; doi:10.1038/s41598-021-01599-3)
Supplement: Supplementary file 1 — Supplementary Information. [file 41598_2021_1599_MOESM1_ESM.pdf]

Supplementary information to:

Unraveling the stepwise maturation of the yeast telomerase including a Cse1 and Mtr10 mediated quality control checkpoint

Anna Greta Hirsch[1], Daniel Becker[2], Jan-Philipp Lamping[1] and Heike Krebber[1*]

[1] Abteilung für Molekulare Genetik, Institut für Mikrobiologie und Genetik, Göttinger Zentrum für Molekulare Biowissenschaften (GZMB), Georg-August Universität Göttingen, Göttingen, Germany

[2] Current address: Philipps-Universität Marburg, Klinik für Dermatologie und Allergologie, Baldingerstraße, D-35043 Marburg

[*]Correspondence: heike.krebber@biologie.uni-goettingen.de

Supplementary Tables

Supplementary Table 1. Yeast strains used in this study. Related to Figures 1-6

Number	Genotype	Source
HKY36	*MATα ura3-52 leu2Δ1 his3Δ200*	[1]
HKY37	*MATα ura3-52 leu2Δ1 his3Δ200 srp1-31*	[2]
HKY46	*MATa ura3-52 lys2-301 ade2 mtr10-1*	[3]
HKY208	*MATα ura3-52 ade2-101 his3-11,15, trp1-Δ901 cse1-1*	[4]
HKY316	*MATa ura3-52 leu2Δ1 trp1Δ63 MTR10-9xMyc-TRP1*	This study
HKY380	*MATa his3Δ1 leu2Δ0 met12Δ0 ura3Δ0 npl3::KanMX4*	Euroscarf
HKY644	*MATα ade2, his3, leu2, trp1, ura3 mex67::HIS3*<br>*pUN100-mex67-5 (LEU2, CEN)*	[5]
HKY1028	*MATα his3Δ1; leu2Δ0; lys2Δ0; ura3Δ0 rrp6::kanMX4*	Euroscarf
HKY1073	*MATα his3Δ1; leu2Δ0; met15Δ0; ura3Δ0; yKu70::kanMX4*	Euroscarf
HKY1079	*MATα his3Δ1; leu2Δ0; met15Δ0; ura3Δ0; RAP1-GFP::HIS3MX6*	[7]
HKY1093	*MATα his3Δ1; leu2Δ0; met15Δ0; ura3Δ0; CDC13-GFP::HIS3MX6*	[7]
HKY1193	*Tgs1::KanMX4/Tgs1::KanMX4*<br>*his3Δ1 / his3Δ1; leu2Δ0 / leu2Δ0; ura3Δ0 / ura3Δ0; lys2Δ0 / LYS2; MET15 / met15Δ0*	Euroscarf
HKY1293	*MATα ura3-52 lys2-801 trp-Δ1 his3-Δ200 leu2-Δ1 tlc1-Δ::HIS*	[6]
HKY1277	*MATa his3Δ1 leu2Δ0 met15Δ0 ura3Δ0 MTR10-GFP:HIS3MX6*	[7]
HKY1353	*MATa ura3-52 mex67::HIS3 xpo1::TRP1*<br>*pUN100 (CEN LEU2) mex67-5 xpo1-1::HIS3*	[8]
HKY1596	*MATa his3Δ1 leu2Δ0 met15Δ0 ura3Δ0 CSE1-GFP:HIS3MX6*	[7]
HKY1642	*MATa ura3-52 lys2-801 ade2-Δ trp1-Δ63 his3-Δ200 leu2-Δ1 smb::KAN*<br>*pGal-TRP1-SmB*	[9]
HKY1645 (*smb smd1*)	*MATa ura3-52 lys2-801 ade2-Δ trp1-Δ63 his3-Δ200 leu2-Δ1 smb::KAN smd1::LEU2*	[9]
HKY1689	*MATα rrp6::kanMX4 mex67::HIS3*<br>*pUN100-mex67-5 (LEU2, CEN)*	[10]
HKY1776	*MATa mtr10:kanMX4*<br>*lys ura leu his*	This study
HKY1799	*MATa his3Δ1 leu2Δ0 met15Δ0 ura3Δ0 CBP20-GFP:HISMX6*	Euroscarf
HKY1815	*MATa ura leu Pop1-GFP:HisMX6*	Euroscarf
HKY2087	*MATα his ura KAN cse1-1 mtr10:kanMX4*	This study
HKY2093	*MATa ura cse1-1 Pop1-GFP:HisMX6*	This study
HKY2101	*MATa leu2Δ1 ura3-52 MTR10-9xMyc-TRP1 Pop1-GFP:HisMX6*	This study
HKY2153	*MATα ura Pop1-GFP:HisMX6 mex67::HIS3*<br>*pUN100-mex67-5 (LEU2, CEN)*	This study
HKY2259	*MATα; ; ura3-52, ade2-101, trp1-Δ901cse1-RAP1-GFP::HIS3MX6*	This study
HKY2261	*MATα; ; ura3-52, ade2-101, trp1-Δ901cse1-CDC13-GFP::HIS3MX6*	This study

Supplementary Table 2. Plasmids used in this study. Related to Figures 1-6.

Number	Genotype	Name
pHK88	CEN URA3	[11]
pHK206	CEN URA CSE1	This study
pHK765	CEN URA GFP-Npl3	[12]
pHK1469	CEN URA SmB-GFP	[13]
pHK1483	CEN URA GFP-POP1	[14]
pHK1589	URA3 EST1-(Gly)6-(myc)12	[15]
pHK1606	CEN URA pAdh-Est1-GFP	This study

Supplementary Table 3. Oligonucleotides used in this study. Related to Figures 1-4, 6. Forward primer (fw) and reverse primer (rev).

Number	Sequence	Name
HK1002	5'-TGCTAAGGCTGTCGGTAAGG-3'	*Tdh1* fw
HK1003	5'-TCAGAGGAGACAACGGCATC-3'	*Tdh1* rev
HK1384	5'-GCGGAAGGAACCGTGTGTTC-3'	*TLC1* immature fw
HK1385	5'-GAAGCCTACCATCACCACACC-3'	Internal *TLC1* fw
HK1386	5'-ACAGCGCTTAGCACCGTCTG-3'	Internal *TLC1* rev
HK1539	5'-DIG-CCACCACACACACCCACACCC-3'	5' DIG labeled CA – Telomeric repeat probe
HK1598	5'-GGCCCCAGGTAAGAAAGTCG-3'	RPL8 fw
HK1599	5'-GAAGGTTTCGGCAGCGGTG-3'	RPL8 rev
HK1738	5'-TGCAAACTCCTTGGTCACAC-3'	U1 snRNA (snR19) fw
HK1739	5'-CCAGGCAGAAGAAACAAAGG-3'	U1 snRNA (snR19) rev
HK1761	5'-CY3-GCGCACACACAAGCATCTACACTGACACCAGCATACTCGAAATTCTTTGG-CY3-3'	*TLC1* probe 1
HK1789	5'-CY3-CGATAAGATAGACATAAAGTGACAGCGCTTAGCACCGTCTGTTTGC-CY3-3'	*TLC1* probe 2
HK1790	5'-CY3-CCTACTCGTATTTTTCTCTGTCACATCGTTCGATGTACGGGGCACATTTGG-CY3-5'	*TLC1* probe 3
HK2154	5'-CCAGAACAATCCGTACACAAGG-3'	*Hem15* fw
HK2155	5'-GCAATTGTCTTCTGATACTTAGCAC-3'	*Hem15* rev
HK2859	5'-CAGCTTTACAGATCAATGGC-3'	U5 snRNA (snR7-L) fw
HK2860	5'-TATGGCAAGCCCACAGTAA-3'	U5 snRNA (snR7-L) rev
HK3089	5'-AGTTACGCTAGGGATAACAGGG-3'	21S rRNA fw
HK3090	5'-TGACGAACAGTCAAACCCTTC-3'	21S rRNA rev
HK3513	5'-ACGCGCGATTTCTACAATAC-3'	*TLC1* immature rev

Supplementary references:

1. Winston F, Dollard C, Ricupero-Hovasse SL. Construction of a set of convenient Saccharomyces cerevisiae strains that are isogenic to S288C. *Yeast (Chichester, England)* **11**, 53-55 (1995).

2. Loeb JD, Schlenstedt G, Pellman D, Kornitzer D, Silver PA, Fink GR. The yeast nuclear import receptor is required for mitosis. *Proceedings of the National Academy of Sciences of the United States of America* **92**, 7647-7651 (1995).

3. Liu Y, Guo W, Tartakoff PY, Tartakoff AM. A Crm1p-independent nuclear export path for the mRNA-associated protein, Npl3p/Mtr13p. *Proceedings of the National Academy of Sciences of the United States of America* **96**, 6739-6744. (1999).

4. Taura T, Krebber H, Silver PA. A member of the Ran-binding protein family, Yrb2p, is involved in nuclear protein export. *Proceedings of the National Academy of Sciences of the United States of America* **95**, 7427-7432 (1998).

5. Segref A*, et al.* Mex67p, a novel factor for nuclear mRNA export, binds to both poly(A)+ RNA and nuclear pores. *The EMBO journal* **16**, 3256-3271 (1997).

6. Lubin JW, Tucey TM, Lundblad V. The interaction between the yeast telomerase RNA and the Est1 protein requires three structural elements. *RNA (New York, NY* **18**, 1597-1604 (2012).

7. Huh W-K*, et al.* Global analysis of protein localization in budding yeast. *Nature* **425**, 686-691 (2003).

8. Brune C, Munchel SE, Fischer N, Podtelejnikov AV, Weis K. Yeast poly (A) -binding protein Pab1 shuttles between the nucleus and the cytoplasm and functions in mRNA export Yeast poly (A) -binding protein Pab1 shuttles between the nucleus and the cytoplasm and functions in mRNA export. *Rna-a Publication of the Rna Society*, 517-531 (2005).

9. Bordonne R. Functional characterization of nuclear localization signals in yeast Sm proteins. *Molecular and cellular biology* **20**, 7943-7954 (2000).

10. Zander G*, et al.* mRNA quality control is bypassed for immediate export of stress-responsive transcripts. *Nature* **540**, 593-596 (2016).

11. Sikorski RS, Hieter P. A system of shuttle vectors and yeast host strains designed for efficient manipulation of DNA in Saccharomyces cerevisiae. *Genetics* **122**, 19-27 (1989).

12. Hackmann A, Gross T, Baierlein C, Krebber H. The mRNA export factor Npl3 mediates the nuclear export of large ribosomal subunits. *EMBO reports* **12**, 1024-1031 (2011).

13. Becker D, Hirsch AG, Bender L, Lingner T, Salinas G, Krebber H. Nuclear Pre-snRNA Export Is an Essential Quality Assurance Mechanism for Functional Spliceosomes. *Cell reports* **27**, 3199-3214 e3193 (2019).

14. Gill T, Aulds J, Schmitt ME. A specialized processing body that is temporally and asymmetrically regulated during the cell cycle in Saccharomyces cerevisiae. *The Journal of cell biology* **173**, 35-45 (2006).

15. Tucey TM, Lundblad V. Regulated assembly and disassembly of the yeast telomerase quaternary complex. *Genes & development* **28**, 2077-2089 (2014).

Supplementary Fig. 1

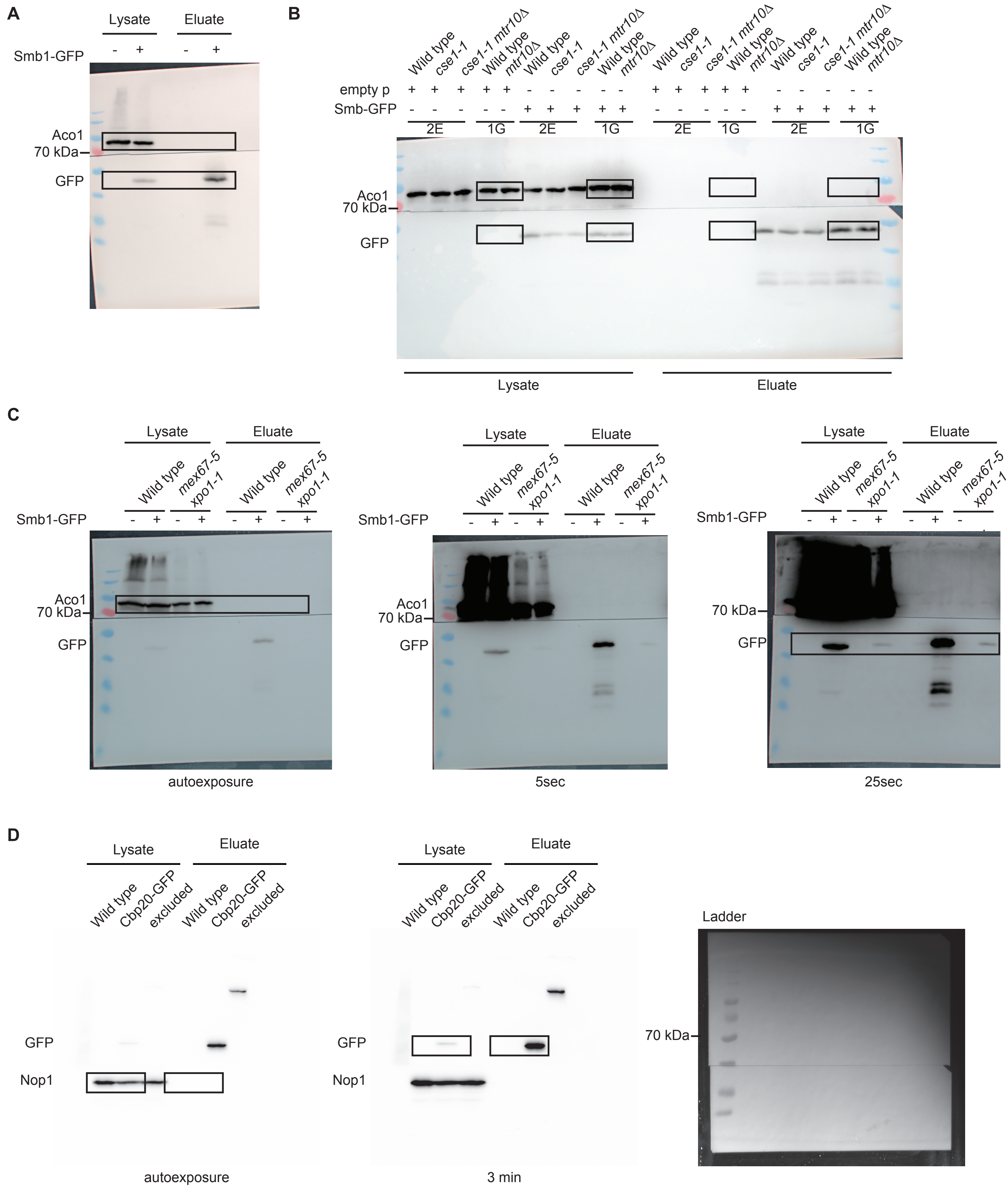

Supplementary Figure 1. Original western blots of Figure 1. The PageRuler Prestained (Thermo Scientific #26616) was used as Ladder.
(A) Original whole western blot shown. For the final figure, photoshop was used to crop the indicated bands (solid black lines) and the bands were placed in Figure 1A.
(B) Original whole western blot shown. For the final figure, photoshop was used to crop the indicated bands (solid black lines) and the bands were placed in Figure 1G and 2E respectively.
(C) Original whole western blot with different exposure times is shown. For the final figure, photoshop was used to crop the indicated bands (solid black lines) and the bands were placed in Figure 1C.
(D) Original whole western blot with different exposure times is shown. For the final figure, photoshop was used to crop the indicated bands (solid black lines) and the bands were placed in Figure 1J.

Supplementary Fig. 2

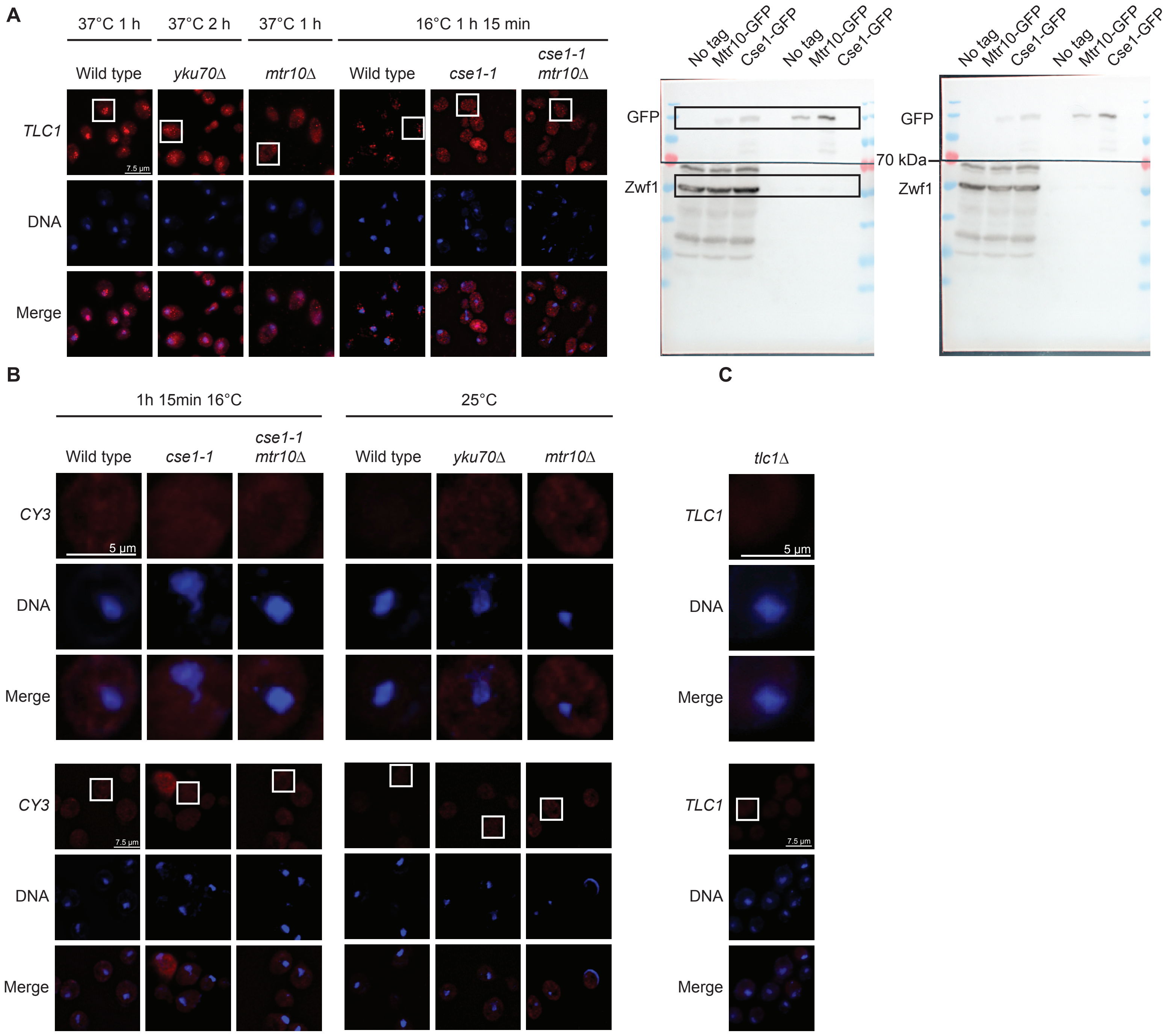

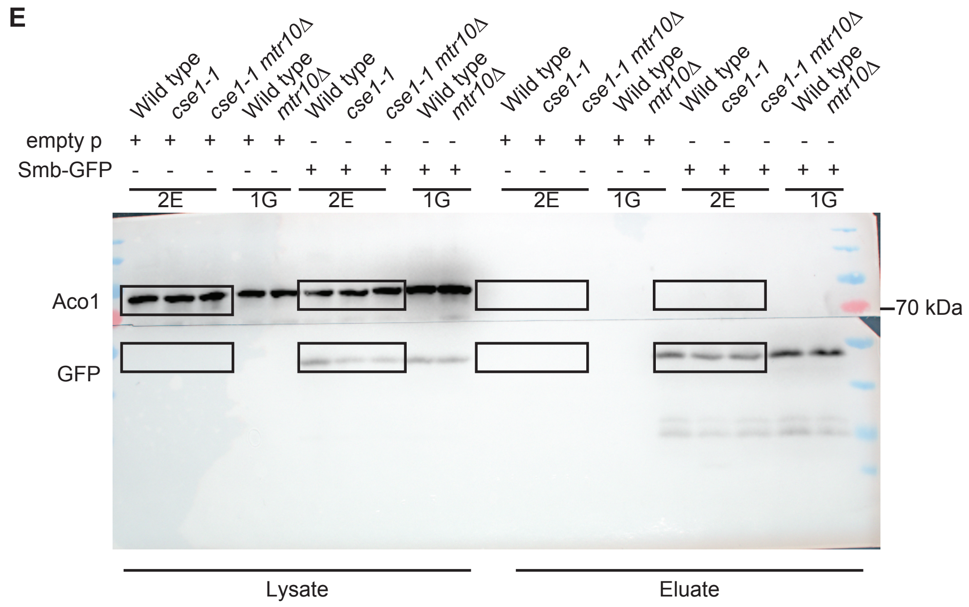

Supplementary Figure 2.
(A) *TLC1* mis-localizes to the cytoplasm in *CSE1* mutants. FISH-experiment for *TLC1* (red) is shown in the indicated strains that were shifted to their non-permissive temperatures. Overview of several cells is shown of which the framed ones are depicted in Figure 2A. *n= 3.*
(B) No probe control of FISH-experiments for *TLC1* is shown in the indicated strains that were shifted to their non-permissive temperatures.
(C) Negativ control of the FISH-experiments for *TLC1* (red) is shown the in the *tlc1Δ* strain.
(D) Original whole western blot shown is shown with high and low exposure time. For the final figure, photoshop was used to crop the indicated bands (solid black lines), and the bands were placed in Figure 2C. The PageRuler Prestained (Thermo Scientific #26616) was used as Ladder.
(E) Original whole western blot shown. For the final figure, photoshop was used to crop the indicated bands (solid black lines), and the bands were placed in Figure 2E and 1G respectively. The PageRuler Prestained (Thermo Scientific #26616) was used as Ladder.

Supplementary Fig. 3

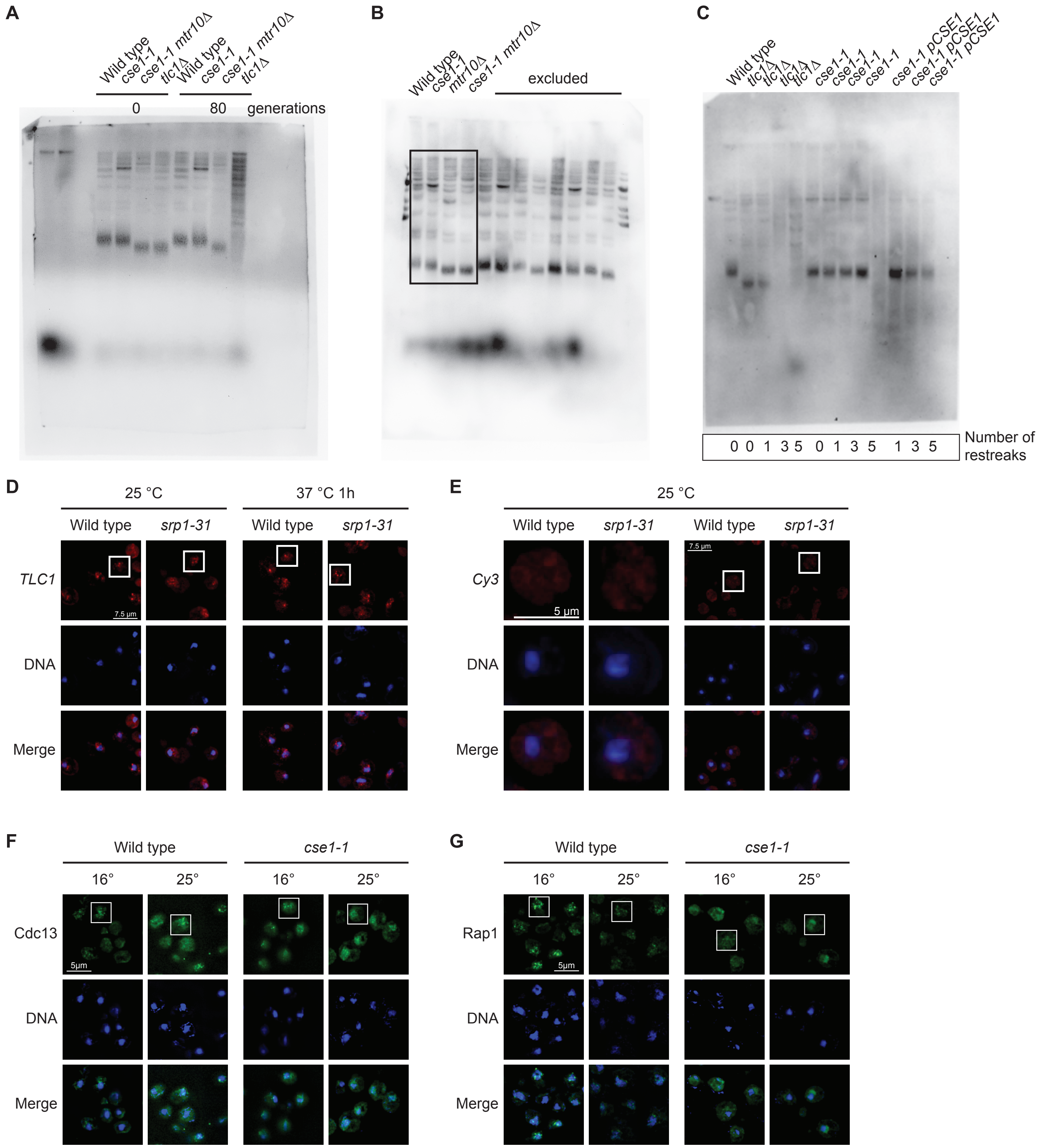

Supplementary Figure 3.
(A) Whole southern blot is shown. XhoI digested genomic DNA of the indicated strains was used for the southern blot. The chromosome ends were detected with a digoxygenin labeled probe. Photoshop was used
to adjust the levels so that the bands were more apparent. The bands were placed in Figure 3B.
(B) (A) Whole southern blot is shown. XhoI digested genomic DNA of the indicated strains was used for the southern blot. The chromosome ends were detected with a digoxygenin labeled probe. Photoshop was used
to adjust the levels so that the bands were more apparent and the cropped area (solid black lines) is shown in Figure 3C.
(C) Whole southern blot is shown. XhoI digested genomic DNA of the indicated strains was used for the southern blot. The chromosome ends were detected with a digoxygenin labeled probe. Photoshop was used
to adjust the levels so that the bands were more apparent. The bands were placed in Figure 3D.
(D) *TLC1* localization is not altered in the *srp1-31* mutant. FISH experiments show no mis-localization of *TLC1* in *srp1-31* after shift to 37 °C for 1h. Overview of several cells is shown of which the framed ones are depicted in (Fig. 3F) *n= 3.*
(E) No probe control of FISH-experiments for *TLC1* is shown in the *srp1-31* mutant.
(F) Cdc13 localization is not altered in the *cse1-1* mutant. Immunofluorescence experiments with an antibody against GFP show no mis-localization of Cdc13 in *cse1-1* after shifting the cells to 16 °C for 1h and 15min. Overview of several cells is shown of which the framed ones are depicted in (Fig. 3G) *n= 3.*
(G) Rap1 localization is altered in the *cse1-1* mutant. Immunofluorescence experiments with an antibody against GFP show nucleolar mis-localization of Rap1 in *cse1-1* at the permissive temperature and a cytoplasmic mis-localization after shifting the strain to 16 °C for 1h and 15min. Overview of several cells is shown of which the framed ones are depicted in (Fig. 3H) *n= 3.*

Supplementary Fig. 4

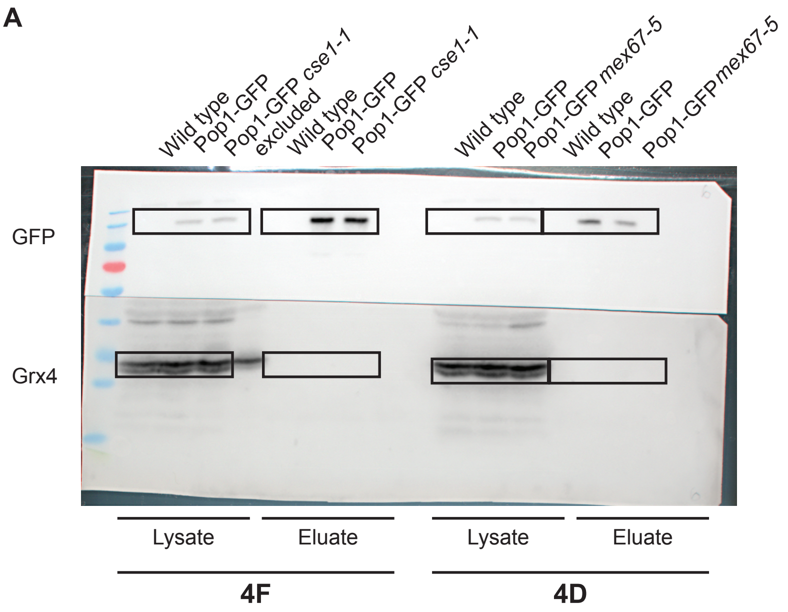

Supplementary Figure 4. Original western blots of Figure 4. The PageRuler Prestained (Thermo Scientific #26616) was used as Ladder

(A) Original whole western blot shown. For the final figure, photoshop was used to crop the indicated bands (solid black lines) and the bands were placed in Fig. 4F and 4D respectively.

Supplementary Fig. 5.1

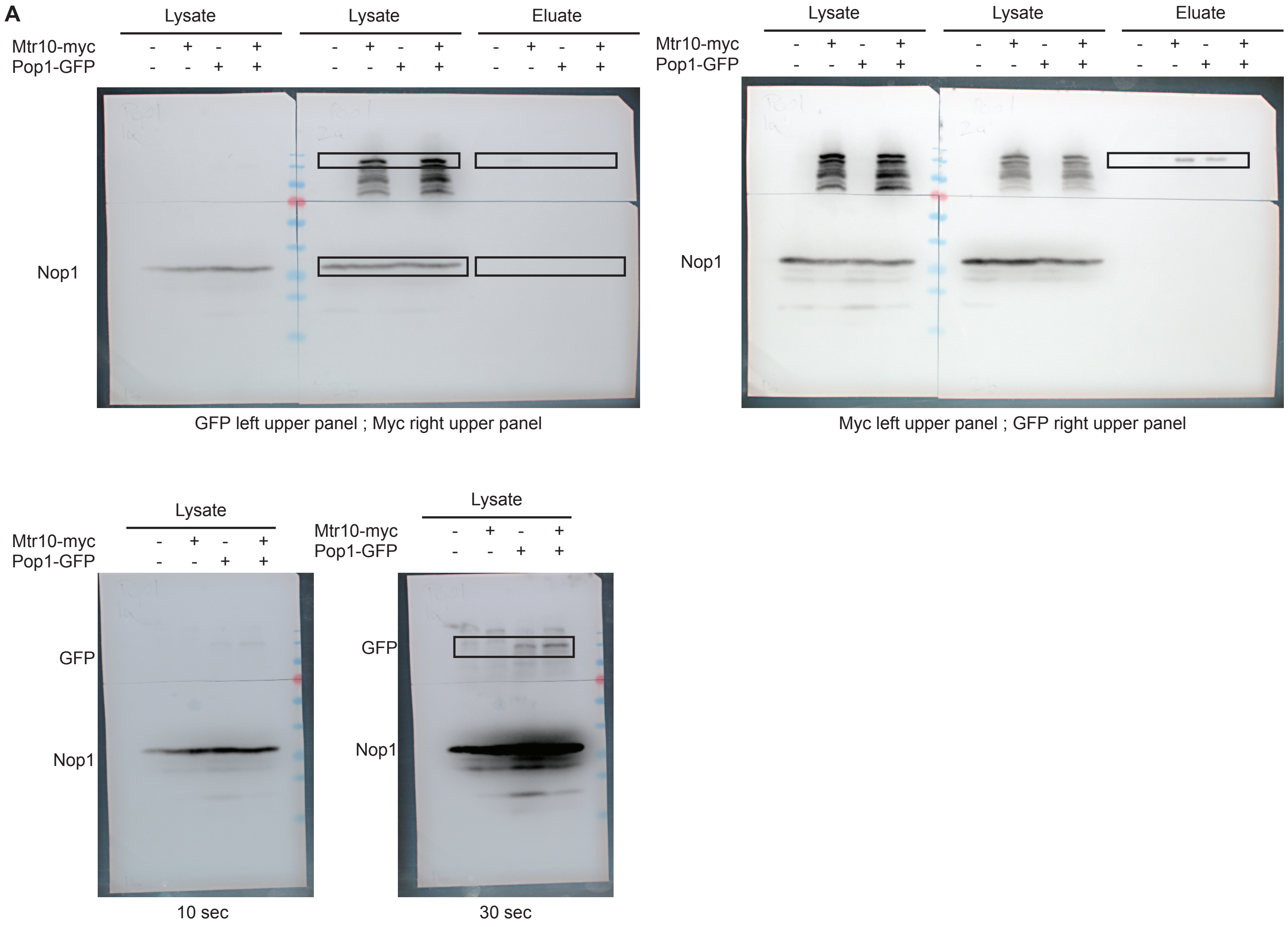

Supplementary Figure 5.1. Original western blots of Figure 5A.
The PageRuler Prestained (Thermo Scientific #26616) was used as Ladder
(A) Original whole western blot shown. The lysates were loaded twice as the tagged proteins have almost identical size. Detection order is indicated underneath the blots. In the Eluate, the first step was to detect the protein to be co-precipitated. Afterwards, pull down detection was carried out. For the final figure, photoshop was used to crop the indicated bands (solid black lines) and the bands were placed in Fig. 5A.

Supplementary Fig. 5.2

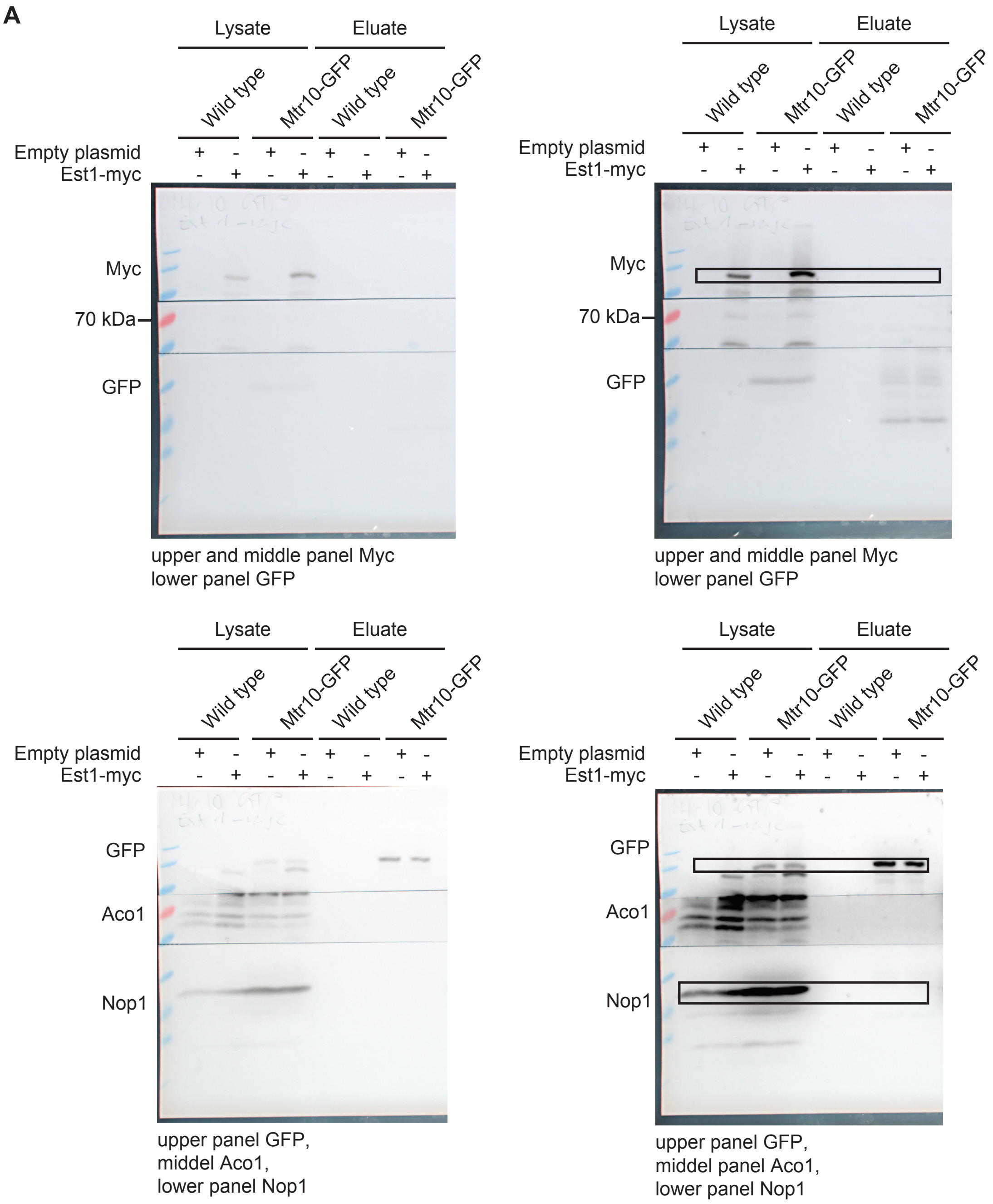

Supplementary Figure 5.2. Original western blots of Figure 5B.
The PageRuler Prestained (Thermo Scientific #26616) was used as Ladder
(A) Original whole western blot shown. Detection order is indicated underneath the blots. In the Eluate, the first step was to detect the protein to be co-precipitated. Afterwards, pull down detection was carried out. For the final figure, photoshop was used to crop the indicated bands (solid black lines) and the bands were placed in Fig. 5B.

Supplementary Fig. 5.3

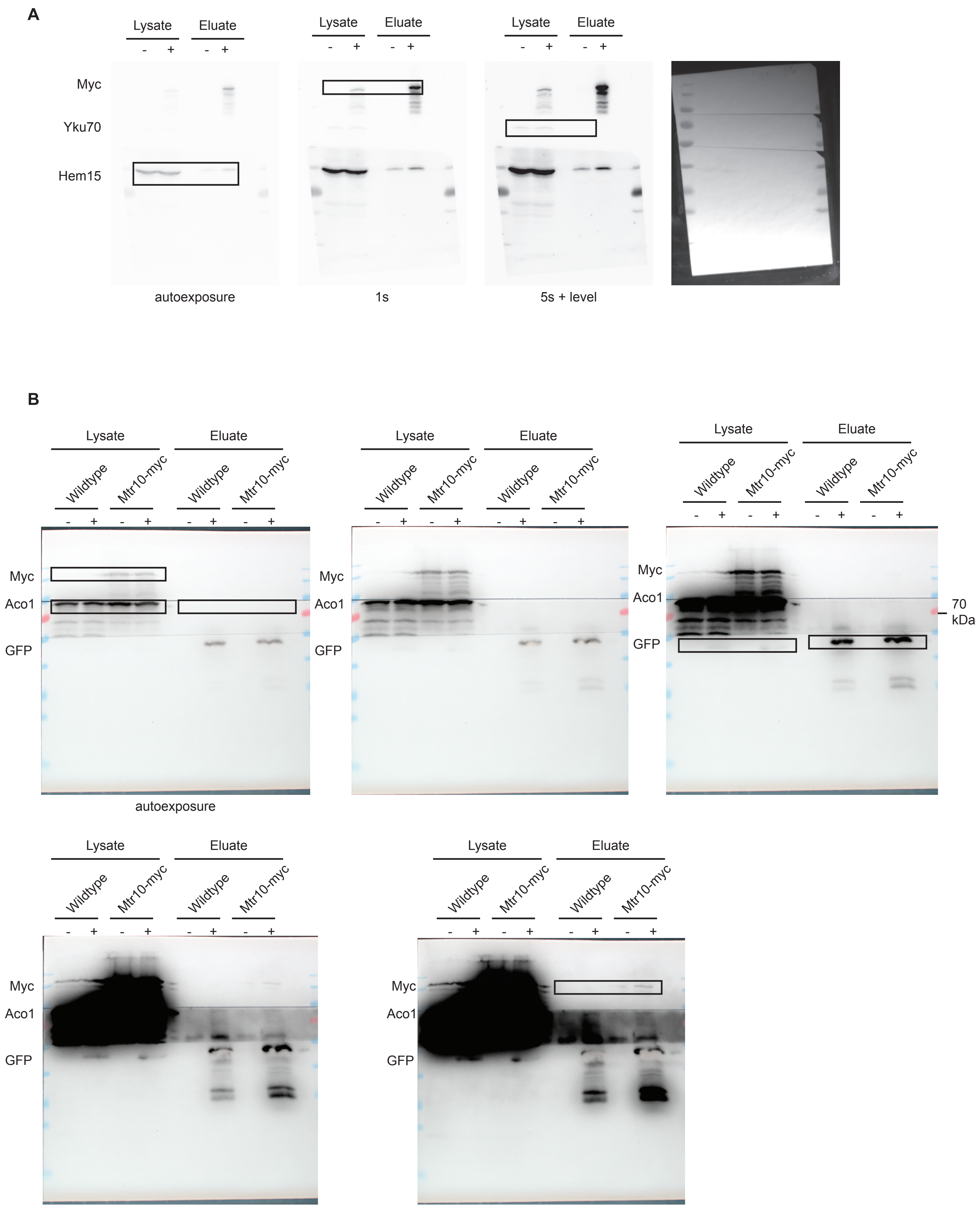

Supplementary Figure 5.3. Original western blots of Figure 5C and 5D.
The PageRuler Prestained (Thermo Scientific #26616) was used as Ladder
(A) Original whole western blot is shown with different exposure times .For the final figure, photoshop was used to crop the indicated bands (solid black lines) and the bands were placed in Fig. 5C.
(B) Original whole western blot is shown with different exposure times. For the final figure, photoshop was used to crop the indicated bands (solid black lines) and the bands were placed in Fig. 5D.

Supplementary Fig. 6

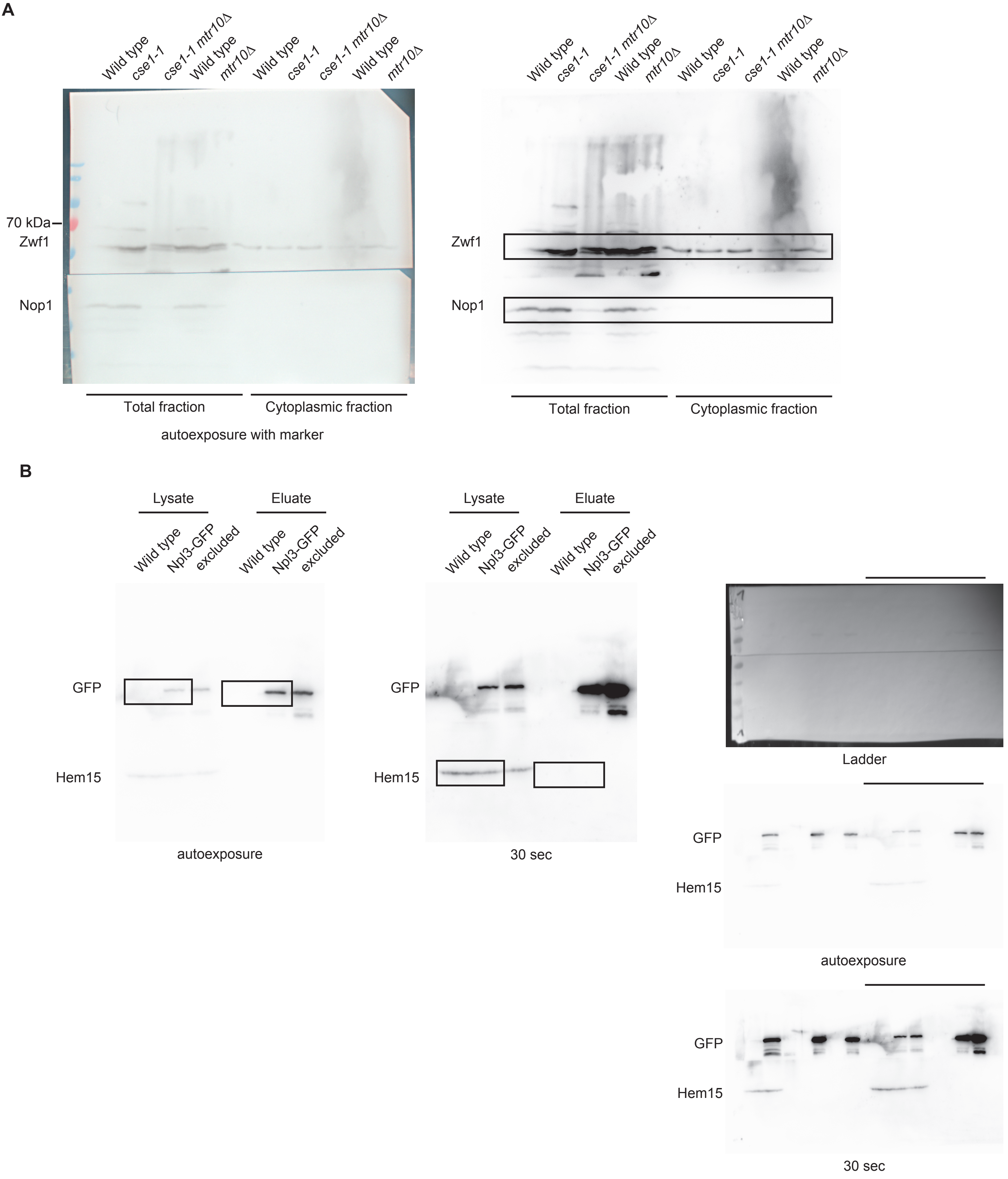

Supplementary Figure 6.
(A) Original whole western blot of a nucleo-cytoplasmic fractionation is shown. For the final figure, photoshop was used to enhance the levels and to crop the indicated bands (solid black lines) and the bands were placed in Figure 6A.
(B) Original whole western blot of an RIP experiment is shown. For the final figure, photoshop was used to crop the indicated bands (solid black lines) and the bands were placed in Figure 6C.
